# On the stability and dynamics of stochastic spiking neuron models: Nonlinear Hawkes process and point process GLMs

**DOI:** 10.1371/journal.pcbi.1005390

**Published:** 2017-02-24

**Authors:** Felipe Gerhard, Moritz Deger, Wilson Truccolo

**Affiliations:** 1 Department of Neuroscience, Brown University, Providence, Rhode Island, United States of America; 2 School of Computer and Communication Sciences and School of Life Sciences, Brain Mind Institute, École polytechnique fédérale de Lausanne (EPFL), Lausanne, Switzerland; 3 Institute for Zoology, Faculty of Mathematics and Natural Sciences, University of Cologne, Cologne, Germany; 4 Institute for Brain Science, Brown University, Providence, Rhode Island, United States of America; 5 Center for Neurorestoration & Neurotechnology, U. S. Department of Veterans Affairs, Providence, Rhode Island, United States of America; Duke University, UNITED STATES

## Abstract

Point process generalized linear models (PP-GLMs) provide an important statistical framework for modeling spiking activity in single-neurons and neuronal networks. Stochastic stability is essential when sampling from these models, as done in computational neuroscience to analyze statistical properties of neuronal dynamics and in neuro-engineering to implement closed-loop applications. Here we show, however, that despite passing common goodness-of-fit tests, PP-GLMs estimated from data are often unstable, leading to divergent firing rates. The inclusion of absolute refractory periods is not a satisfactory solution since the activity then typically settles into unphysiological rates. To address these issues, we derive a framework for determining the existence and stability of fixed points of the expected conditional intensity function (CIF) for general PP-GLMs. Specifically, in nonlinear Hawkes PP-GLMs, the CIF is expressed as a function of the previous spike history and exogenous inputs. We use a mean-field quasi-renewal (QR) approximation that decomposes spike history effects into the contribution of the last spike and an average of the CIF over all spike histories prior to the last spike. Fixed points for stationary rates are derived as self-consistent solutions of integral equations. Bifurcation analysis and the number of fixed points predict that the original models can show stable, divergent, and metastable (fragile) dynamics. For fragile models, fluctuations of the single-neuron dynamics predict expected divergence times after which rates approach unphysiologically high values. This metric can be used to estimate the probability of rates to remain physiological for given time periods, e.g., for simulation purposes. We demonstrate the use of the stability framework using simulated single-neuron examples and neurophysiological recordings. Finally, we show how to adapt PP-GLM estimation procedures to guarantee model stability. Overall, our results provide a stability framework for data-driven PP-GLMs and shed new light on the stochastic dynamics of state-of-the-art statistical models of neuronal spiking activity.

## Author summary

Earthquakes, gene regulatory elements, financial transactions, and action potentials produced by nerve cells are examples of sequences of discrete events in space or time. In many cases, such events do not appear independently of each other. Instead, the occurrence of one event changes the rate of upcoming events (e.g, aftershocks following an earthquake). The nonlinear Hawkes process is a statistical model that captures these complex dependencies. Unfortunately, for a given model, it is hard to predict whether stochastic samples will produce an event pattern consistent with observations. In particular, with positive feedback loops, the process might diverge and yield unrealistically high event rates. Here, we show that an approximation to the mathematical model predicts dynamical properties, in particular, whether the model will exhibit stable and finite rates. In the context of neurophysiology, we find that models estimated from experimental data often tend to show metastability or even unstable dynamics. Our framework can be used to add constraints to data-driven estimation procedures to find the optimal model with realistic event rates and help to build more robust models of single-cell spiking dynamics. It is a first step towards studying the stability of large-scale nonlinear spiking neural network models estimated from data.

## Introduction

Point-process generalized linear models (PP-GLMs) have become an important approach in the statistical modeling of neurophysiological responses from single nerve cells and their interactions in neural circuits [1–8]. A specific class of PP-GLMs are nonlinear Hawkes processes [9, 10]. In this case, each action potential (spike) modulates the firing intensity of the neurons in the future. Nonlinear Hawkes PP-GLMs can capture the major canonical dynamics of single neurons [11–13] and as phenomenological models avoid the many issues that arise in the specification of biophysically detailed neuronal models [14, 15]. In this way, nonlinear Hawkes PP-GLMs are also important phenomenological models for the simulation and study of large-scale neuronal network models of brain function. However, nonlinear Hawkes PP-GLMs also lead to non-renewal point process spike train statistics because contributions to the intensity from many previous spikes can accumulate over arbitrary time scales [16, 17]. This raises the question of whether such models will produce stable, stationary dynamics in simulations, or whether firing rates will diverge or settle into unphysiological rates depending on a specified absolute refractory period.

For the linear Hawkes point process model, stability can be assessed by calculating the integral of the spike-history kernel, i.e., the effect that each spike has on subsequent activity of the same cell [18–20]. However, for the prevalent nonlinear case, no such practical criterion is currently available. Main stability results established by Brémaud and Massoulié are too restrictive for our applications [9, 21]. Furthermore, model parameters are typically estimated from data using maximum-likelihood methods [2]. For linear autoregressive processes, it is well known that maximum-likelihood estimates can lead to unstable dynamics [22, 23]. We expect this to be even more severe in nonlinear models.

Here, we first show that PP-GLMs estimated from physiological data might not generate spike train realizations that match even simple statistics such as mean firing rates of the original data. Instead, firing rates tend to diverge to the maximum firing rate that is allowed in the presence of an absolute refractory period. Firing patterns like this would typically be considered unphysiological. This can happen in spite of the models passing commonly used goodness-of-fit tests based on, for example, the time-rescaling theorem [24, 25].

To address the above stability issues, we propose an approximative framework to derive stochastic stability conditions for PP-GLMs. For a stochastically stable point process, the state of the point process stochastically evolves in time, but can be described by a stable and time-invariant distribution of “states” (sample paths), resulting in a stationary point process [9]. In contrast, a process that is not stochastically stable may show similar stochastic dynamics for some time, but eventually its state may diverge and never return. In neural point processes, this scenario is typically associated with a divergence of the firing rate. In case of an actual divergence there is no stationary distribution of states. Note, however, that when considering absolute refractory periods, the divergence of the firing rate and associated internal states of the point process are limited. Firing with an inter-event interval equal to the refractory period is nonphysiological, and the state distribution in this mode of firing is singular. We call this a diverged state in slight deviance from the usual terminology. Our use of the term stochastic stability, however, is in line with the definition from stochastic dynamical systems, but generalized to stochastic point processes (see also [9 Remark 4]).

Our approach to derive stability conditions for PP-GLMs is based on a recently developed mean-field theory of neural dynamics [26, 27]. The approach relies on the following steps. First, we use a quasi-renewal (QR) approximation that decomposes spike history effects into the contribution of the last spike and an average of the conditional intensity function (CIF) over all spike histories prior to the most recent spike. Second, after truncation of a moment-based expansion, this decomposition leads to a tractable expression for the approximated CIF. Third, under stationarity conditions, fixed points can be derived as self-consistent solutions of an integral equation, which correspond to expected steady-state firing rates of the neuron. Fourth, depending on the number and stability of these fixed points, each single-neuron model can be unambiguously classified into one of three types: stable, divergent, or fragile. The latter corresponds to metastability which results from stochastic fluctuations perturbing the dynamics in the presence of multiple stable fixed points and when the upper fixed point corresponds to a stable but unphysiologically high firing rate. Fluctuations around the low-rate fixed point of the network dynamics predict an expected time horizon until rates will converge to the high-rate state. This expected time metric can be used to estimate the probability of firing rates to remain finite for a given time period. Examination of the stability of the fixed points and how it depends on the shape of the spike-history filter not only determines the stability of the stochastic dynamics, but also leads to general stability constraints on PP-GLM parameters.

In the following sections, we present our framework to assess the stability of a specific neuron model in detail. We validate the QR approximation in comparison to results of numerical simulations for a large range of artificial neuron models that are neurophysiologically plausible. We then apply the method to real-world data sets. Finally, we demonstrate and discuss how parameter estimation procedures could be adapted to ensure stability of estimated models.

As stated above, stability of neuron models is particularly important when numerical simulations are desired or spike trains are to be generated from the model. Generated spike trains can be used to assess model goodness-of-fit and to perform forecasting of neural activity over longer time scales. Our results are a first step towards ensuring stability for recurrently connected neural network models. These models can be put in the framework of multivariate nonlinear Hawkes models, and our classification framework conceptually translates to the multivariate case. Stable (or stabilized) neuron models play an important role in the field of computational neuroscience, especially in the simulation of large-scale models of brain function. They are also important in neuroengineering, where neuron models are embedded in hybrid and closed-loop applications [28, 29].

## Results

### Estimated data-driven PP-GLMs can diverge, despite passing goodness-of-fit tests

**Fig 1:**
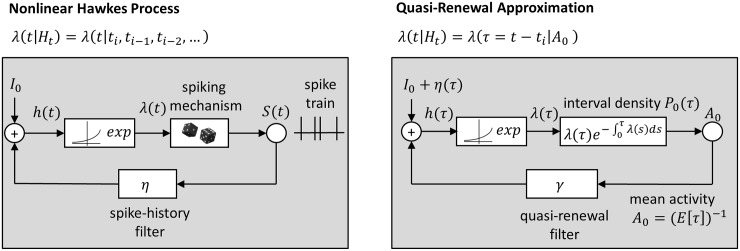
Schematic overview of the nonlinear Hawkes process and the quasi-renewal (QR) approximation. The quasi-renewal approximation can be used to semi-analytically obtain steady-state firing rates of general, nonlinear Hawkes processes (PP-GLMs). Left: In the nonlinear Hawkes process, the conditional intensity of the point process, $\lambda (t|H_{t})$, is a function of the whole spiking history (see Eq (3)). It is modeled as a nonlinear function (here, an exponential function) of a linear convolution of the previous spike history with a spike-history filter *η*(*s*) plus a constant offset *I*_0_. The dependence of the instantaneous firing rate on all previous spikes results in a non-renewal process. There are no closed-form solutions for even the first-order statistics of general, nonlinear Hawkes processes. Right: In the quasi-renewal approximation, the conditional intensity is modeled as a combination of the effect of the most recent spike *t*_*i*_ and a term involving the average over the whole spike history before the most recent spike (see Eq (5)). This term includes the average firing activity in the past *A*_0_ ≡ *A*(*t* − *s*), which is filtered with the quasi-renewal filter *γ*(*s*) and added to the spike-history filter *η*(*τ*) of the most recent spike at *t* − *τ*. This predicts the instantaneous inter-spike interval density *P*_0_(*τ*) from which the steady-state firing rate can be obtained as the inverse of the expected inter-spike interval *E*[*τ*]. The self-consistent solutions for which an assumed average history of *A*_0_ leads to an equivalent predicted steady-state rate are fixed points of the transfer function defined in the quasi-renewal approximation.

The nonlinear Hawkes process is a point process model that is commonly used to describe neurophysiological responses. It defines the conditional intensity function (instantaneous firing rate) of a neuron as a nonlinear function of previous spiking activity (Fig 1A):

$$\lambda \left(t|H_{t}\right)=\phi \left(h(t)\right),$$ (1)

where $H_{t}$ denotes the spiking history up to time *t* and *ϕ*(*x*) is a non-negative nonlinear function. The term *h*(*t*) consists of a constant offset *I*_0_ and a convolution of the spike train *S*(*t*) with (temporal) spike-history kernels or filters *η*(*s*):

$$h\left(t\right)=I_{0}+\left[\eta *S\right]\left(t\right)=I_{0}+\sum_{k=1}^{K}\eta \left(t-t_{k}\right),$$ (2)

where the $\left\{t_{k}\right\}\in H_{t}$ correspond to the previous spike times (see “Materials and Methods” for details). Based on both theoretical and empirical arguments [2, 7], we set *ϕ*(*x*) = exp(*x*) to arrive at:

$$\lambda \left(t|H_{t}\right)=\exp\left(I_{0}+\sum_{k=1}^{K}\eta \left(t-t_{k}\right)\right)=c\exp\left(\sum_{k=1}^{K}\eta \left(t-t_{k}\right)\right),$$ (3)

with $c=e^{I_{0}}>0$.

Every previous spike contributes a spike-history kernel, and effects of all previous spikes accumulate. This leads to, in general, a non-renewal point process model. The model parameters that describe the kernel *η*(*s*) and the baseline firing rate *c* can be estimated using maximum-likelihood optimization within the framework of generalized linear models (GLMs) [2, 4].

**Fig 2:**
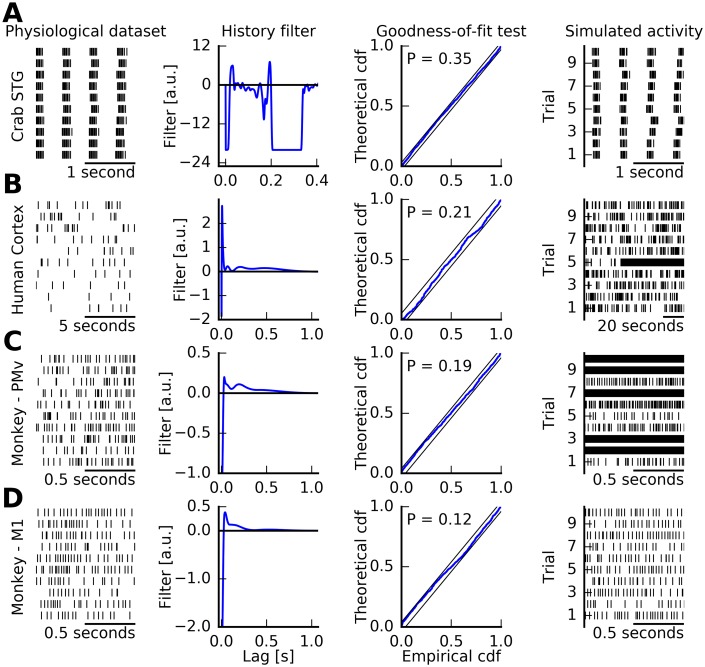
Point-process models estimated from physiological data can pass common goodness-of-fit tests, but simulated activity may diverge. (A) Neurons in the stomatogastric ganglion (STG) of the crab show rhythmic bursts of spike patterns. Each line shows a random 2-second segment of the data from one neuron aligned to the first spike of a burst. The spike-history filter is estimated following the procedure in [6]. The neuron model passes commonly used goodness-of-fit tests, such as those based on the time-rescaling theorem [24, 25]. Here, the Kolmogorov-Smirnov test is shown for rescaled inter-spike intervals to come from an exponential distribution with unit mean. The null hypothesis that observed spikes are coming from the estimated model is not rejected (*P* > 0.05). When sampling spike trains from the model, the model regenerates the rhythmic, bursty activity that is qualitatively matched to the training data. (B) Similar analysis for single-unit activity from neocortical recordings in a person with pharmacologically intractable focal epilepsy [30]. Each line corresponds to a random ten-second segment of spontaneous activity during interictal periods, i.e., outside seizures. The estimated spike-history filter shows a refractory period and an excitatory rebound. The model passes commonly used goodness-of-fit tests (*P* > 0.05). When stochastic samples are generated from the model, spiking activity diverges to a periodic firing pattern at the maximally allowed frequency given the absolute refractory period (here, 2 ms). For some sampled realizations, this divergence can happen very early in the simulated trial (e.g., trial 5). Therefore, simulated activity from the model is unphysiological. It does not match statistics of the spike train in the training data (mean firing rate, inter-spike interval statistics) despite passing the goodness-of-fit test. (C, D) Additional examples of single-unit activity from monkey cortex, areas PMv and M1 [31, 32]. Each line represents a steady-state movement preparation period preceding visual cues leading to execution of reach and grasp actions. Although spike-history filters appear typical in both examples, and goodness-of-fit tests are passed, simulated activity diverges into unphysiological firing rates in one case (first example) and remains physiological in the other.

As stated earlier, these point-process GLMs (PP-GLMs) were recently shown to be able to describe all major canonical dynamics of single neurons [11–13] and, thus, can serve as a canonical class of mathematically tractable models to describe general single-neuron spiking activity. For example, in [6], we analyzed spiking data from the stomatogastric nervous system of the crab. Neurons that are part of the pyloric network fire in stereotypical, rhythmic activity patterns (Fig 2A). Estimated PP-GLMs from this physiological dataset pass common goodness-of-fit tests such as based on residual analysis or the time-rescaling theorem. We created stochastic realizations of spike trains based on the model. These spike trains reproduce the observed burst pattern of the training data, and in a complete network simulation, the relative phases of the overall pyloric rhythm [6].

However, such simulations of spike trains from data-driven PP-GLMs do not always result in physiological spiking patterns. For example, when estimating PP-GLMs from single-unit data recorded from the neocortex of human epileptic patients [30], goodness-of-fit tests are generally passed, but simulated activity tends to diverge to unphysiologically high firing rates. One data set and estimated model are shown in Fig 2B.

In a more comprehensive analysis, we fitted PP-GLMs to spiking data from neurons recorded from motor-related cortical areas in the monkey [31]. We restricted data to a one-second steady-state movement preparation period of the trial. This period was roughly stationary since it did not include, by design, firing rate transients driven by sensory stimuli or movement execution. For 35 out of the 99 data-driven models, we find that simulated spike trains have finite divergence times. Some of these models diverge in simulations even when goodness-of-fit tests are passed. We show two examples in Fig 2C and 2D. Qualitatively similar results are obtained for all other models.

Instability is also observed when the simulation is performed using a nonlinearity that grows less rapidly than the exponential. We generated stochastic realizations of spike trains using two additional nonlinearities (a linear rectifier, and *f*(*x*) = log(1 + *e*^*x*^), a smooth interpolation between an exponential and linear function, S1 Fig). Both functions are globally dominated by the exponential function (see S1A Fig). Potentially, nonstable behavior could be observed when simulating with the exponential nonlinearity but not with the two less rapidly accelerating nonlinearities. However, we find that even in these cases, firing rates diverged for the same data sets as presented in Fig 2B and 2C.

In summary, while PP-GLMs estimated from data may serve well in encoding and decoding analysis that require one-step spike prediction conditioned on actually observed spike history and may pass goodness-of-fit tests, they tend to be poor generative models because of the lack of stochastic stability. The use of PP-GLMs as generative models, however, is essential when statistical analyses of spike trains generated by the model are required, or when long-term prediction of future spiking states in single-neuron and neuronal networks is used in neural decoding or closed-loop interventions.

To our knowledge, the stability of PP-GLMs estimated from data has not been systematically examined before. In the next sections, we will develop a framework to assess the dynamics and stability of stochastic spiking neuron models.

### A framework to assess stability and dynamics of stochastic spiking neuron models

PP-GLMs have post-spike filters which typically make the spike train probability depend on many previous spikes. These dynamics are in contrast to the conditional intensity function of a renewal point process which depends only on the very last spike time. Therefore, PP-GLMs are generally not renewal processes. When assessing dynamics and stability of PP-GLMs, we are interested in the behavior of the corresponding firing rates. For such general PP-GLMs, however, there are no closed-form solutions for even simple statistical features, such as expected mean firing rates or second-order statistics. Here, to obtain estimates of such statistical features for a given nonlinear Hawkes process, we employ an approximation based on a recently introduced quasi-renewal approximation [26, 27].

The quasi-renewal approximation (Fig 1B) consists of approximating the (non-renewal) PP-GLM by a process which is nearly a renewal-process (hence, “quasi-renewal”) that depends on the last spike time and on the average firing rate in the past [26]. Specifically, we consider the steady-state conditional intensity $\lambda_{0}\left(t,\hat{t}\right)$ at time *t* as the average intensity over all possible spike histories that share the most recent spike at time $\hat{t}$:

$$\begin{array}{l} \begin{array}{ccc} \lambda_{0}\left(t,\hat{t}\right) & = & {\langle \lambda (t|H_{t})\rangle }_{S(t<\hat{t})}\\ & = & c\;\text{exp}\left(\eta \left(t-\hat{t}\right)\right){\langle \text{exp}\left(\left[\eta *S\right]\left(t\right)\right)\rangle }_{S(t<\hat{t})}. \end{array} \end{array}$$ (4)

The first term explicitly models the effect of the most recent spike only, and the second term represents the average of the spiking activity prior to the time of the last spike in the steady-state regime. It can be approximated by (see “Materials and Methods” for details):

$${\langle \exp\left(\left[\eta *S\right]\left(t\right)\right)\rangle }_{S(t<\hat{t})}\approx \exp\left\{A_{0}\int_{t-\hat{t}}^{\infty }\left(\underset{\gamma (u)}{\underset{︸}{e^{\eta (u)}-1}}\right)du\right\},$$ (5)

with *γ*(*u*) = *e*^*η*(*u*)^ − 1 for the exponentiated spike-history kernel. Here, *A*_0_ is the steady-state firing rate of the process. Intuitively, the convolution of the actual spike train *S*(*t*) with *η*(*s*) is replaced by the convolution of a homogeneous Poisson process spike history of intensity *A*(*t* − *u*) ≡ *A*_0_ with an effective filter *γ*(*u*) (Fig 1B). Since the convolution is applied to a constant *A*_0_, the term reduces to a product of *A*_0_ and the integral of *γ*(*u*) with the lower bound dependent on $\tau =t-\hat{t}$. Using this approximation, we obtain a quasi-renewal CIF by combining Eqs (4) and (5). Given the QR-CIF, Eq (4), we can then as for ordinary renewal processes, derive the steady-state survivor function $S_{0}$ as:

$$S_{0}\left(\tau \right)=\exp\left\{-\int_{0}^{\tau }\lambda_{0}\left(u\right)du\right\}.$$ (6)

$S_{0}$ then yields the steady-state probability density *P*_0_ of the inter-spike intervals:

$$P_{0}\left(\tau \right)=S_{0}\left(\tau \right)\lambda_{0}\left(\tau \right).$$ (7)

The inverse of the expected inter-spike interval must equal the firing rate *f* which thus is an implicit function of *A*_0_ through Eq (5):

$$f\left(A_{0}\right)={\left[\int_{0}^{\infty }\tau \;P_{0}\left(\tau \right)d\tau \right]}^{-1}.$$ (8)

In effect, the QR theory derives a transfer function *f*(*A*_0_) > 0 that links an assumed average spike history to a predicted firing rate. Assuming stationarity, *f*(*A*_0_) has to match *A*_0_ which leads to a fixed-point equation. Intersections of *f*(*A*_0_) with the identity correspond to expected fixed points of the dynamics. Stable fixed points in the quasi-renewal approximation predict steady-state firing rates of nonlinear Hawkes processes (PP-GLMs).

**Fig 3:**
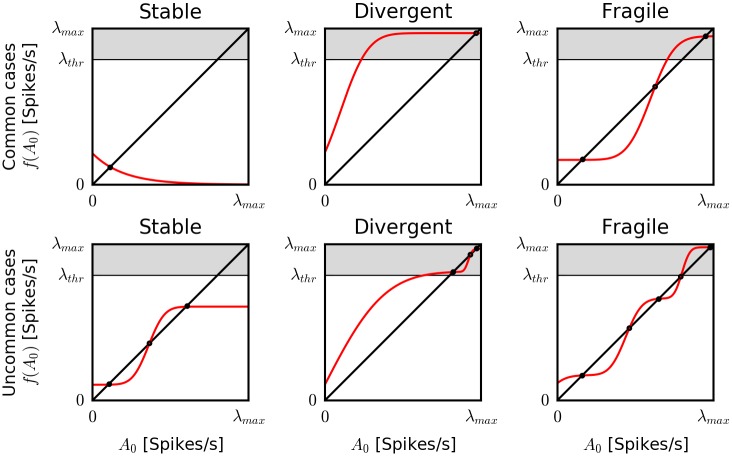
Stochastic dynamics of nonlinear Hawkes PP-GLMs endowed with absolute refractory periods: Classification based on transfer functions in the quasi-renewal approximation. The quasi-renewal approximation provides a predicted firing rate of a neuron model *f*(*A*_0_) based on an assumed average firing rate in the past *A*_0_ (see “Materials and Methods”). This defines an iterative equation whose fixed points represent the steady-state firing rates. A qualitative classification of the dynamical behaviors is based on the location and stability of the fixed points. Note that throughout the study, we are assuming that there exists an absolute refractory period. Thereby, the maximum firing rate of any model is limited by a maximal firing rate *λ*_*max*_. We define a steady-state firing rate to be unphysiological if it exceeds *λ*_*thr*_ = 0.9 × *λ*_*max*_ (gray area). Given an absolute refractory period, there always exists at least one stable fixed point. If it is the only one and below *λ*_*thr*_, the model is classified as stable (top, left). If the only stable fixed point is above *λ*_*thr*_, the model is divergent (top, center). If there are two stable fixed points, one above and one below *λ*_*thr*_, the model is classified as “fragile” (metastable), indicating that the (physiological) low-rate fixed point is only transiently stable. Expected divergence times E[*T*_div_] will depend on the distance between the fixed points. To provide a complete classification framework, we also need to consider the case of two or more stable fixed points, although the latter case seems to be rarely encountered in our experience. In case of two or more stable fixed points below the threshold, the model is classified as stable (bottom, left). Its dynamics is predicted to be multi-stable with steady-state rates fluctuating around two fixed points. If all stable fixed points lie above the threshold the model is considered “divergent” (bottom, center). Any case for which there are multiple stable fixed points both below and above *λ*_*thr*_ are considered “fragile” (bottom, right).

We first show how the number and stability of fixed points of the derived transfer function *f*(*A*_0_) for the nonlinear Hawkes process endowed with an absolute refractory period can be used to classify the dynamical behavior of the single-neuron model (Fig 3):

- **No fixed point**: If there is no stable fixed point (i.e., *f*(*A*_0_) > *A*_0_ for all *A*_0_), activity will diverge after a finite transient. However, this cannot happen in the presence of an absolute refractory period *τ*_ref_. The maximal firing rate of the neuron is bounded by *λ*_*max*_ = 1/*τ*_ref_. In that case, there is always at least one stable fixed point. Throughout the rest of the paper, we assume the existence of such an absolute refractory period and set *τ*_ref_ = 2 ms.
- **One stable fixed point**: Any perturbation in the firing rate will eventually decay back to the steady-state rate. If the steady-state firing rate predicted by the fixed point is close to the mean firing rate of the training data, the rate is considered physiological and we classify the neuron model as “stable” (Fig 3 top, left). Conversely, if the fixed point is above *λ*_*thr*_ = 0.9 × *λ*_*max*_, we define here the rate as unphysiologically high and classify the model as “divergent” (Fig 3 top, center).
- **Two stable fixed points**: More than one stable fixed point leads to multi-stable dynamics. Due to the continuity of the transfer function, the first fixed point is necessarily stable, followed by an unstable fixed point, and so on, in alternating fashion. In general, in stochastic multi-stable dynamics, activity will remain around one state for some time before fluctuations drive it towards a different stable fixed point (metastability). Depending on the location of the stable fixed points, such multi-stable activity can be classified as “stable” (both fixed points smaller than *λ*_*thr*_; Fig 3 bottom, left) or “divergent” (both fixed points larger than *λ*_*thr*_; Fig 3 bottom, center). Interesting dynamics emerges when one fixed point is within the range of physiological rates and the other one is close to the “divergent”, maximal rate (Fig 3 top, right). In that case, if activity is initialized in the lower state, it will remain in the lower state for a finite time before switching to the unphysiological, high-rate state. We call these metastable dynamics “fragile” to emphasize that such models may produce realistic firing rates in simulations when initialized in the low state but will ultimately escape the stable fixed point and diverge to unphysiological high-firing activity. By E[*T*_div_], we denote the expected time to transition from the low-rate state to the high-rate state for the first time. Expected divergence times E[*T*_div_] will depend on the distance between the fixed points and the location of the unstable fixed point.
- **Three or more stable fixed points**: Dynamics can be classified analogously to the previous cases based on how many fixed points are above and below *λ*_*thr*_ (Fig 3 bottom, right). However, more than two stable fixed points seem rare in our datasets and simulations. Although they might be constructed by a deliberate choice of the spike-history filter *η*(*s*), all models that we estimated from physiological data turned out to have at most two stable fixed points.

**Fig 4:**
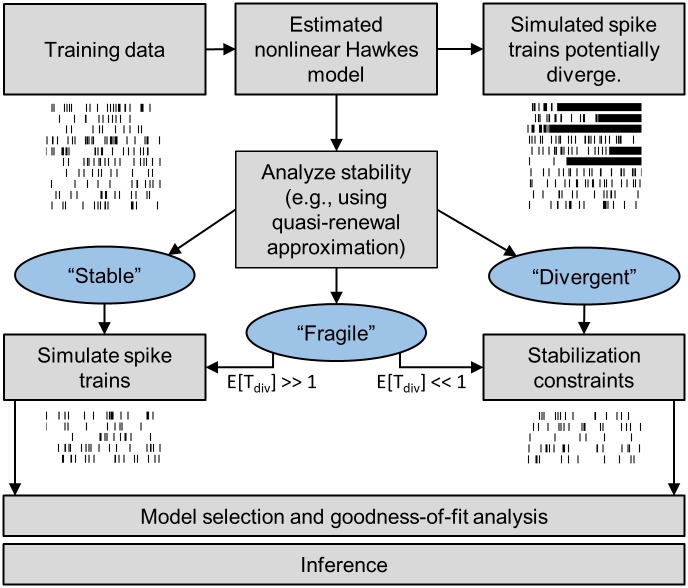
A framework to assess stability and dynamics of stochastic spiking neuron models. Stability of models estimated from physiological data is analyzed using the quasi-renewal approximation. When stable models are desired for sampling or simulations, stability constraints can be included in model estimation. Three types of dynamics can be distinguished: First, “stable” models have steady-state firing rates that are in the physiological range. Spike trains can be safely generated from the model. Second, “divergent” models have a steady-state firing rate that is very close to the maximally allowed firing rate. In this case, stabilization constraints can be added to the maximum-likelihood optimization problem to constrain the feasible parameter space to non-divergent models. Finally, model dynamics can be classified as “fragile”, indicating metastable dynamics. While there is a steady-state firing rate at physiological firing rates, there are additional steady-state rates at unphysiological high rates. A simulation that is started with physiological initial conditions may remain in the low-rate regime for a while, but will ultimately visit the unphysiological rate. The framework may provide an estimate of the expected escape or “divergence” time, E[*T*_div_]. Depending on E[*T*_div_], the model can be effectively treated as “stable” or “divergent” based on the typical time scales that would be relevant for simulation. In any of the three cases, the model (or its stabilized variant) is evaluated based on standard model selection and goodness-of-fit tests before any inference is made.

Overall, the above classification of the qualitative stochastic dynamics suggests a general framework to assess stability and dynamics of stochastic spiking neuron models (Fig 4). In the particular case of data-driven models, training data are used to estimate parameters of a nonlinear Hawkes model (PP-GLM) through (regularized) maximum-likelihood optimization. As shown above, we find empirically that simulating spike trains from these models often yields unphysiological spiking patterns, and firing rates may diverge (Figs 2 and 4, top). We can use the quasi-renewal approximation to analyze the stability of the estimated neuron model. This approximation predicts the dynamics of the neuron model and distinguishes three qualitatively different dynamical behaviors.

Stable or fragile models with high expected divergence times can be safely used to generate stochastic samples from the model. For divergent models or fragile models with low expected divergence times, stabilization constraints can be added to the maximum-likelihood optimization problem to constrain the feasible parameter space to non-divergent models. In any of the three cases, the model (or its stabilized variant) is evaluated based on standard model selection and goodness-of-fit tests before any inference is made (Fig 4, bottom).

In the next sections, we present the application of this framework to simulated and real data to demonstrate its validity and utility in modeling electrophysiological responses. First, we demonstrate the proposed method for PP-GLMs that have a spike-history filter that consists of either a single exponential or a sum of two exponentials before moving on to filters estimated from neurophysiological data.

### Predicted stability reflects simulation outcomes for exponential spike-history filters

We start with the analysis of a simple PP-GLM with a spike-history filter given by a single exponential and an absolute refractory period. The complete model is given by:

$$\begin{array}{ccc} \lambda (t|H_{t}) & = & ce^{\left[\eta *S](t\right)},\\ \eta (s) & = & J\theta \left(s\right)e^{-s/\tau }+J_{\text{ref}}\theta \left(s\right)\theta \left(\tau_{\text{ref}}-s\right), \end{array}$$

with Heaviside function *θ*(*x*) and parameters *τ*_ref_ = 2 ms, *J*_ref_ = −∞ (−10^12^ in numerical simulations), *τ* = 20 ms, and amplitude *J*.

**Fig 5:**
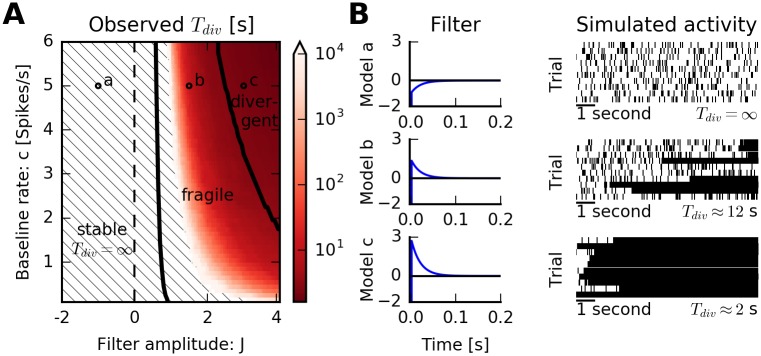
Predicted stability reflects simulation outcomes: Exponential spike-history filter. (A) Spike trains are simulated from a nonlinear Hawkes model with baseline firing rate *c* and an auto-history kernel *η*(*s*) = *J* exp(−*s*/*τ*) with amplitude *J*, time constant *τ* = 0.02 s, and absolute refractory period *τ*_ref_ = 2 ms. The QR approximation qualitatively predicts three classes of dynamics (separated by thick lines). Color indicates average divergence times estimated from simulations. In the dashed region, no finite divergence times were observed. (B) An example of a spike-history filter for each of the dynamics is shown together with a spike raster of simulated activity. Purely inhibitory filters produce stable dynamics (top, *J* = −1, *c* = 5 s^−1^). For fragile models, after an episode of irregular firing, dynamics switches into a tonic firing mode close to the limit frequency 1/*τ*_ref_ (middle, *J* = 1, *c* = 5 s^−1^). For divergent models, the only stable fixed point is close to the limit frequency (bottom, *J* = 3, *c* = 5 s^−1^). After a brief transient, the model switches to the tonic firing mode at limit frequency. All dynamics observed in simulations are in accordance with the prediction from the QR approximation.

We scanned the two-dimensional parameter space given by the amplitude *J* of the filter and the baseline firing rate *c* (Fig 5A; −2 ≤ *J* ≤ 4 with 121 equally spaced samples and 0.1 ≤ *c* ≤ 6.0 *s*^−1^ with 60 equally-spaced samples). The QR approximation predicts three regimes of dynamical activity: For slightly positive and negative kernel amplitude *J*, the model is stable. Indeed, in simulations, we observe finite and stable rates (Fig 5B, top row). For higher amplitudes, the dynamics are predicted to be fragile and ultimately divergent (for large *J* and *c*). As expected, the average divergence time estimated from numerical simulations gradually decreases with increasing *J* and *c* (color-coded in Fig 5A). Divergent models are almost instantly diverging (Fig 5B, bottom row), while for fragile models, a whole spectrum of divergence times is observed (Fig 5B, middle row). Within the variance given by the finite number of simulated models, we did not observe any discrepancies between the behavior predicted by the QR approximation and the numerical results.

**Fig 6:**
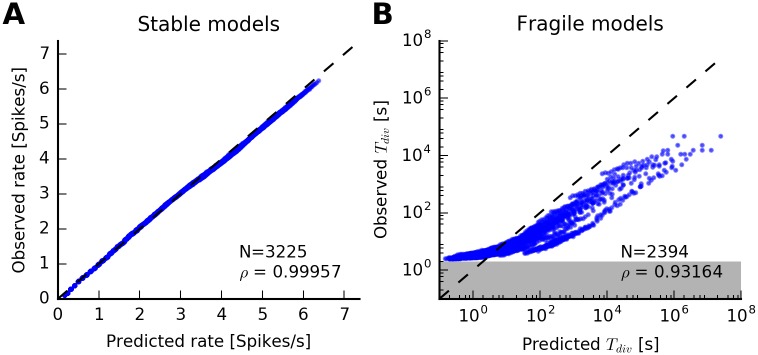
Rate and time to divergence: Exponential spike-history filter. (A) Observed steady-state firing rate versus predicted steady-state firing rate for every model classified as “stable” in Fig 5. Each dot corresponds to a model. Dashed line indicates equality. Linear correlation coefficient is *ρ* = 0.9996. (B) Observed versus predicted divergence times for all models classified as “fragile” in Fig 5. Note the logarithmic axes. The QR approximation provides an approximation of the divergence times. The divergence time of a simulation was defined as the end of the first two-second interval in which the average rate exceeded *λ*_*thr*_. Therefore, estimated divergence times cannot be below 2 s (gray area).

For all models of the parameter range that were classified as stable, we compared the predicted steady-state firing rate to the one observed in numerical simulations (Fig 6A). In this case, the QR approximation provides an excellent prediction of mean firing rates (Pearson’s correlation coefficient *ρ* > 0.999).

A major feature of the QR approximation is to predict (an upper bound on) the expected divergence times for fragile models. In practice, this is relevant for model sampling via simulation where it is important to classify fragile models as “effectively stable” or divergent (see Fig 4). For high firing rates close to $A_{0}=\tau_{\text{ref}}^{-1}$, the regular spike train with inter-spike intervals around *τ*_ref_ is the only possible spike train realization. Therefore, one way to estimate E[*T*_div_] is to consider periodic spike histories with different frequencies that would lead to self-sustained periodic firing at maximal rate with high probability (see “Materials and Methods” for details).

Fig 6B compares predicted versus observed divergence times for all fragile models. The predicted *T*_div_ provide an upper bound on the observed divergence times. The divergence time of a simulation was defined as the end of the first two-second interval in which the average rate exceeded *λ*_*thr*_. For this reason, estimated divergence times cannot be below 2 s (Fig 6B, gray area). Therefore, small estimated divergence times do not obey the predicted bound. However, there seems to be a reasonable (power-law) dependence between predicted and observed *T*_div_ (Pearson’s correlation coefficient *ρ* = 0.925).

We now look at more complex PP-GLMs to test the validity of our proposed framework. We consider spike-history filters consisting of a sum of two exponentials with amplitudes *J*_*r*_, *J*_*a*_, and corresponding time constants *τ*_*r*_ and *τ*_*a*_:

$$\begin{array}{ccc} \lambda (t|H_{t}) & = & ce^{\left[\eta *S](t\right)},\\ \eta (s) & = & J_{r}\theta \left(s\right)e^{-s/\tau_{r}}+J_{a}\theta \left(s\right)e^{-s/\tau_{a}}+J_{\text{ref}}\theta \left(s\right)\theta \left(\tau_{\text{ref}}-s\right). \end{array}$$

Depending on the signs of the amplitudes, this model resembles many plausible single-neuron behaviors: *J*_*r*_ < 0 indicates a relative refractory period beyond the absolute 2 ms refractory period while *J*_*r*_ > 0 promotes bursty dynamics. Similarly, *J*_*a*_ ≠ 0 can be interpreted as inhibitory or facilitating adaptation (e.g., spike-frequency adaptation [16, 17, 33]).

**Fig 7:**
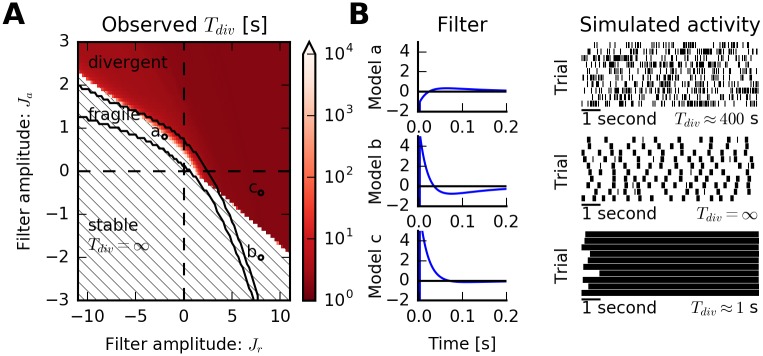
Predicted stability reflects simulation outcomes: Sum-of-exponentials spike-history filter. (A) Spike trains are simulated from a nonlinear Hawkes model with fixed baseline *c* = 5 s^−1^ and an auto-history kernel consisting of two exponentials with amplitudes *J*_*r*_, *J*_*a*_, and corresponding time constants *τ*_*r*_ = 0.02 s and *τ*_*a*_ = 0.1 s. Observed divergence times for simulated spike trains are color-coded (same scale as in Fig 5). In the dashed region, no finite divergence times were observed. (B) Auto-history kernels for three different parameter values (models a, b and c), displaying irregular, bursty and divergent dynamics, respectively, consistent with the prediction of the QR-approximation except for model b (see main text).

We evaluated models on a wide range of combinations of amplitudes *J*_*r*_ and *J*_*a*_ (Fig 7A; −11 ≤ *J*_*r*_ ≤ 11 with 100 equally spaced samples and −3 ≤ *J*_*a*_ ≤ 3 with 75 equally-spaced samples), for *τ*_*r*_ = 20 ms and *τ*_*a*_ = 100 ms, respectively, and for fixed *c* = 5 *s*^−1^. As expected, negative and positive but small values of *J*_*r*_ and *J*_*a*_ lead to stable dynamics. For a narrow band, models are expected to be fragile (Fig 7B, top row). This observation is consistent with divergence times estimated from numerical simulations. Finally, larger values of either *J*_*a*_ or *J*_*r*_ lead to divergent models, although in an asymmetric way (Fig 7A and 7B, bottom row). Qualitatively similar results are obtained for other values of the baseline *c* (S2 and S3 Figs).

Estimated divergence times are generally consistent with the qualitative prediction of the QR approximation with one exception: For multiphasic spike-history filters, i.e. either strongly refractory neurons (*J*_*r*_ ≪ 0) with excitatory rebounds (*J*_*a*_ ≫ 0) or the opposite (*J*_*r*_ ≫ 0 and *J*_*a*_ ≪ 0), the QR approximation predicts divergent models, but simulations indicate that rates remain below the threshold *λ*_*thr*_ to be classified as divergent (Fig 7A, upper left and lower right corners; Fig 7B, middle row). Spike trains generated from models with these parameters tend to produce intermittent bursts. This is a condition for which the quasi-renewal approximation is known to become invalid [26]. The dynamics lead to the divergent state where the model neuron fires initially at maximally allowed firing rate (hence, unphysiological). However, the dynamics escape this high-firing rate fixed point after a finite number of such high-rate bursts, and the activity reverts back to the low-rate state. Averaged over a longer time period, the mean activity stays well below *λ*_*thr*_ and therefore, ${\hat{T}}_{\text{div}}=\infty$ in contrast to the dynamics predicted by the QR approximation. The reason for this discrepancy is that the QR approximation assumes homogeneous, Poisson-like firing prior to the last spike time, while the only way to achieve firing rates close to the maximally allowed rate by the absolute refractory period is to have a highly regular spike train. For highly regular spike histories, the QR approximation does not provide valid fixed points of the dynamics. However, these cases can be captured by an analysis of the regular spiking limit (see “Bursting and the regular spiking limit”) which does not explicitly depend on the QR approximation.

**Fig 8:**
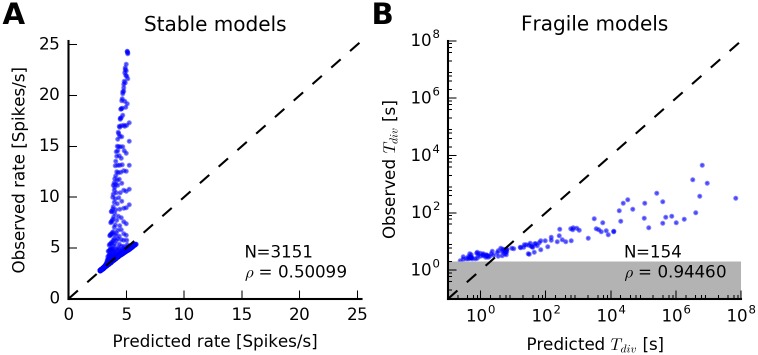
Rate and time to divergence: Sum-of-exponentials spike-history filter. (A) Observed steady-state firing rate versus predicted steady-state firing rate for every model classified as “stable” in Fig 7. Each dot corresponds to a model. Dashed line indicates equality. Linear correlation coefficient is *ρ* = 0.50. While steady-state rates are correctly predicted for most models, discrepancies arise due to limiting assumptions of the QR approximations when *J*_*r*_ ≫ 0 and *J*_*a*_ ≪ 0. However, the qualitative prediction of the dynamics as stable remains correct. (B) Observed versus predicted divergence times for all models classified as “fragile” in Fig 7. Note the logarithmic axes. Dashed line indicates diagonal. The QR approximation provides an approximation of the divergence times. The divergence time of a simulation was defined as the end of the first two-second interval in which the average rate exceeded *λ*_*thr*_. Therefore, estimated divergence times cannot be below 2 s (gray area). The data suggest a power-law dependence between predicted and observed divergence times (Pearson’s correlation coefficient *ρ* = 0.94).

A less severe limitation of the QR approximation is visible in the comparison between predicted and simulated steady-state firing rates for models classified as stable (Fig 8A). While most rates are accurately estimated (points near the diagonal line), very bursty neurons have higher firing rates in simulations than predicted by the QR approximation (stable models with *J*_*r*_ ≫ 0 and *J*_*a*_ ≪ 0). This is due to dependencies beyond the last spike that are ignored in the approximation but are non-negligible for burst firing. Loosely speaking, in this case the QR approximation predicts the rate of isolated spikes and *bursts*, but not the number of total spikes. The burst duration can be predicted from a simple criterion based on the conditional intensity function of the PP-GLM, independent of the QR approximation (see “Bursting and the regular spiking limit”).

Finally, predicted and observed divergence times for all fragile models are well approximated by the QR approximation (Fig 8B) except for small divergence times whose estimation is biased due to the finite time window to detect divergence in numerical simulations.

In summary, the QR approximation yielded remarkably accurate predictions of the dynamical behavior of PP-GLMs for most parameter settings. When the steady-state rate was not accurately predicted for bursty neurons, the qualitative prediction was still consistent with simulations. For extreme parameter values, we observed intermittent burst activity that was incorrectly predicted to be divergent. Although not divergent according to the definition of Fig 3, the resulting spiking pattern would nevertheless be considered unphysiological and undesirable in modeling applications. Thus, in this case, the discrepancy between the QR prediction and the simulation does not play a significant role in practice.

So far, we have studied parametric spike-history filters in the form of a single exponential or sum of two exponential terms. In the next section, we will show that the validity of the QR approximation extends to physiological PP-GLM spike-history filters as they are typically obtained in the context of data-driven model estimation.

### The quasi-renewal approximation predicts stability for complex (physiologically plausible) model parameters

We applied the QR approximation to models estimated from actual neuronal recordings, specifically, multi-electrode single-unit recordings in monkey cortex (see “Materials and Methods”). Of the nonlinear Hawkes PP-GLMs estimated from 99 recorded single units, 11 were predicted to be stable, 86 were predicted as “fragile” with varying degrees of expected divergence times, and 2 were predicted to be divergent.

In all cases, the predictions were consistent with numerical simulations: For all models predicted to be stable, none of the *N* = 48 simulations of length *T* = 1000 s diverged (${\hat{T}}_{\text{div}}=\infty$), and both divergent models showed finite divergence times (${\hat{T}}_{\text{div}}=86\;\text{s}$ and ${\hat{T}}_{\text{div}}=2\;\text{s}$, respectively). Fragile models did not diverge in our simulations of length *T* = 1000 s in 53 out of the 86 cases, while the other maximum-likelihood models diverged with varying degrees of observed divergence times (${\hat{T}}_{\text{div}}=4-45\;000\;\text{s}$).

**Fig 9:**
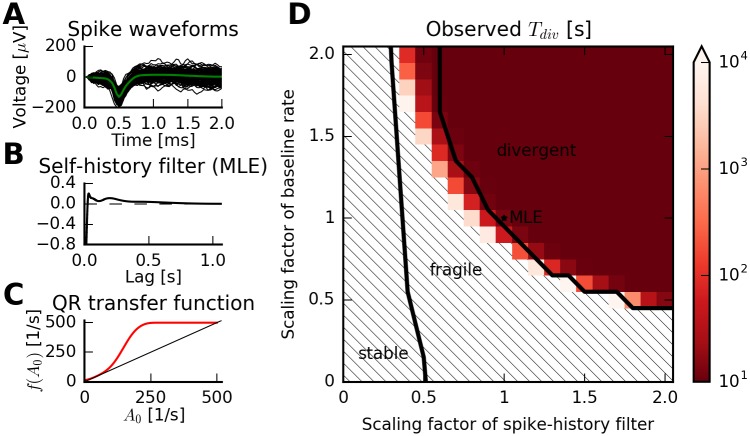
The quasi-renewal approximation predicts stability for complex (physiologically plausible) model parameters. (A) Single-unit activity (SUA) from multi-electrode recordings from monkey cortical area PMv [31] (same unit as in Fig 2C). Spike waveforms are shown (mean waveform in green) and indicate well-sorted SUA. (B) Estimated spike-history kernel using maximum-likelihood estimation. The kernel exhibits a relative refractory period followed by an excitatory rebound. (C) The transfer function predicted by the QR approximation. There is a single stable fixed point at ≈ 500 s^−1^. The model is therefore classified as “divergent”. (D) The QR approximation predicts the maximum-likelihood estimate (MLE) (center dot) to be divergent. Color indicates average divergence times in simulations for variations of the baseline rate *c* (y-axis) and scaled versions of the filter relative to the integral of *e*^*η*(*s*)^ − 1 (x-axis). Thick lines indicate the separation between areas for which the QR approximation predicts stability, fragility, or divergence. Overall, estimated divergence times from simulations agree with the qualitative predictions.

We examined in detail the stability predictions based on the QR approximation for a divergent neuron model in Fig 9. Spike-sorted single-unit activity (Fig 9A) was used to estimate a nonlinear Hawkes process with ten basis functions for the spike-history filter consisting of raised cosines [4, 5]. The resulting maximum-likelihood estimate (MLE) displays a relative refractory period followed by an excitatory rebound (Fig 9B). The corresponding transfer function of the QR approximation shows a single stable fixed point close to the maximally allowed firing rate (Fig 9C). Therefore, this model is classified as divergent.

We then explored the neighboring parameter space by varying the baseline rate parameter *c* and using scaled versions of the MLE spike-history filter. The qualitative predictions (separated by thick lines in Fig 9D) were overall consistent with numerical simulations of the model (Fig 9D). Here, the color scale represents the estimated divergence time in simulations based on 48 independent simulations of *T* = 1000 s each.

In sum, the QR approximation not only predicted correctly the stability of the data-driven neurophysiological models, but also the stability of parameter variations around the MLE model.

### Neuron models can be stabilized with constrained maximum-likelihood estimation

We have shown that fitting PP-GLMs to electrophysiological data can lead to divergent and fragile models (Figs 2 and 9). The QR approximation not only offers a way to predict stability, but also to find stable models. As stated before, stability is an important feature when the goal is to sample from the model or to obtain data-driven models for simulations. Conceptually, we can constrain the parameter search for the maximum-likelihood solution to the parameter space for which the QR approximation predicts stable models. We will now provide a proof of concept of this approach by demonstrating how this constraint can be implemented in practice.

We search for the maximum-likelihood estimate (MLE) under the additional constraint that the model is predicted to be stable by the QR approximation. This can be implemented by minimizing the cost function consisting of the negative log-likelihood of the data plus a penalty term that is infinity whenever the model is predicted to be fragile or divergent and zero otherwise. We use a gradient-free numerical optimization scheme (see “Materials and Methods”). We initialize the parameter values with the unconstrained MLE for which all positive coefficients are set to zero. This corresponds to a non-positive spike-history filter and ensures that the initial evaluation of the cost function is finite. We call the constrained solution the “stabilized MLE”.

**Fig 10:**
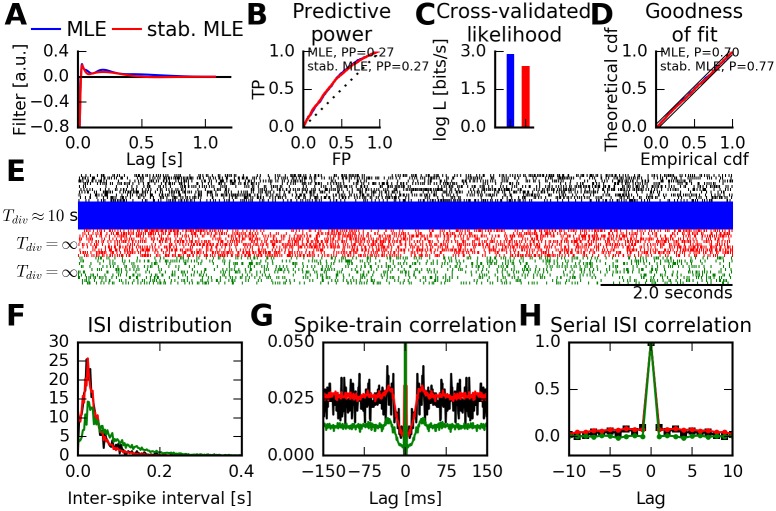
Neuron models can be stabilized with constrained maximum-likelihood estimation. (A) Estimated spike-history kernels using maximum-likelihood estimation (blue) and the constrained (stabilized) maximum-likelihood estimation (red). Data are from single-unit activity (SUA) recordings from monkey cortical area PMv [31]. (B) Power of predicting spiking activity on test data for both the maximum-likelihood estimate (MLE) and the stabilized MLE. The receiver operating characteristic (ROC) curve is shown for predicting spikes in 1 ms time bins with false positive rate (FP, x-axis) and true positive rates (TP, y-axis). Diagonal line indicates chance-level prediction. Predictive power is defined as PP = 2 ⋅ AUC − 1 with AUC being the area under the curve. Perfect spike prediction corresponds to PP = 1. Both models predict spikes equally well. (C) Log-likelihood evaluated on test data. Both MLE and stabilized MLE models preserve information about spike times. Model log-likelihoods (in bits per second) are relative to a homogeneous Poisson process with the correct spiking rate. (D) Kolmogorov-Smirnov test of rescaled inter-spike intervals following the time-rescaling theorem. Both MLE and the stabilized MLE pass the goodness-of-fit test (*P* > 0.05). (E) Recorded spike trains and simulated spike trains from estimated models. Top: Randomly selected 10 s intervals of neural activity part of the training data (black). Simulating from the unconstrained MLE model (blue) leads to quickly diverging firing rates (*T*_div_ ≈ 10 s). Spiking activity from the stabilized MLE (red) remains finite and physiological. Simulating from the MLE with a reset condition after each spike (green, [34]) leads to non-divergent firing rates, but the firing rate of the training data is not matched. (F) Inter-spike interval statistics of real and simulated spiking activity for which rates were non-divergent. Same colors as in (D). The stabilized MLE qualitatively reproduces the training data ISI distribution. (G) Autocorrelation of recorded and simulated activity. (H) Serial ISI correlations of real and simulated spiking activity. The stabilized MLE accurately reproduces correlations in the training data. Simulating the MLE with a reset condition (green) leads to a renewal process, hence vanishing correlations at non-zero lags.

For illustration, in Fig 10A, we use the same data from monkey electrophysiological recordings as in Fig 9. We find that the spike-history filter of the stabilized MLE resembles a regularized version of the (unconstrained) MLE (Fig 10A), i.e., its coefficients are slightly biased towards zero. The MLE itself passes a goodness-of-fit test on training and test data and yields a substantial power in predicting spiking in 1 ms time bins (Fig 10B–10D). However, sampling from the MLE leads to divergent and unphysiological spike trains (Fig 10E). In contrast, sampling from the stabilized model yields firing rates comparable to the training data, and spike trains that are qualitatively similar (Fig 10E).

We quantified similarity of spike train statistics up to second order using the inter-spike interval (ISI) distribution (Fig 10F) and spike train auto-correlation function (Fig 10G). We evaluated the spiking pattern using three metrics (local ISI variability *lv*, shape (log *κ*) and scale parameter (log *α*) of the best-fitting Gamma distribution, following the methodology presented in [35, 36]. Our data from monkey region PMv are consistent with reported values in [36, Table 1, row 15]: *lv*^(data)^ = 0.54 ± 0.18, log *κ*^(data)^ = 0.80 ± 0.38, log *α*^(data)^ = 3.16 ± 0.26 (mean ± standard deviation over segments of 20 consecutive ISIs). Metrics for spike trains generated from the stabilized MLE model are well aligned to those of the physiological data: *lv*^(stab.MLE)^ = 0.66 ± 0.22, log *κ*^(stab.MLE)^ = 0.69 ± 0.35, and log *α*^(stab.MLE)^ = 3.26 ± 0.26. In addition, the spike trains generated from the stabilized MLE also reproduce the serial correlations between consecutive ISIs in the physiological data (Fig 10H).

Although this was not a direct optimization criterion, the stabilized MLE is almost as good in predicting spiking activity in 1 ms time bins conditioned on observed spiking history as the MLE (Fig 10B) and scores only marginally worse on the goodness-of-fit tests (Fig 10D). The cross-validated log-likelihood of the stabilized model is within 80% of the log-likelihood score of the MLE (2.43 bits/s versus 2.90 bits/s relative to the prediction of a homogeneous Poisson process with correct spiking rate, Fig 10C).

Spike train statistics were not shown for the MLE because physiological spike trains could not be obtained. However, we explored the possibility of a simple modification in the simulation procedure for the MLE solution that guarantees stability of the generated spike train. In the “reset condition” [34], the conditional intensity of the point process at time *t* is not calculated using the original CIF (Eq (3)) but instead using only the spike-history effect stemming from the most recent spike at $\hat{t}:\;\lambda \left(t|H_{t}\right)=c\exp\left(\eta \left(t-\hat{t}\right)\right)$. This yields a renewal process with guaranteed finite rate in simulations.

We found that models simulated with the reset condition do produce finite firing rates and spike trains that look plausible on first sight (Fig 10D). However, the firing rate and autocorrelation function are not matched with the training data. Most notably, neither ISI shape (Fig 10F and 10G, green line; *lv*^(reset)^ = 0.72 ± 0.21, log *κ*^(reset)^ = 0.51 ± 0.31, log *α*^(reset)^ = 2.57 ± 0.20) nor ISI correlations are reproduced due to the renewal property of the modified model (Fig 10H, green line).

## Discussion

We have presented a framework to predict the stability and dynamics of a general class of stochastic neural point process models, specifically nonlinear Hawkes processes and point process GLMs. This framework is based on a quasi-renewal approximation of the exact conditional intensity function model. The assessment of stability can serve as an additional goodness-of-fit test along with other approaches, such as tests based on the time-rescaling theorem. We have also shown that simulated activity from point-process models estimated from neurophysiological data tends to exhibit unphysiologically high firing rates. This behavior results from lacking stochastic stability of the estimated PP-GLMs. When sampling and simulations based on these PP-GLMs is desired, our framework can be used to derive stability constraints which can be included into standard parameter estimation techniques such as (regularized) maximum-likelihood optimization. Furthermore, our framework provides a way to determine and classify the qualitative types of stochastic dynamics exhibited by PP-GLMs, specifically nonlinear Hawkes processes with absolute refractory periods.

Empirically, we have shown that many point-process GLMs estimated from neurophysiological data tend to show fragile or even divergent dynamics. Several reasons could explain this finding. One potential scenario is that of the actual recorded neural dynamics being close to instability. Even assuming a correctly specified model, finite data will lead to finite variance in the parameter sets estimated via maximum likelihood. If the true parameters are close to the boundary between stability and divergence, the estimated MLE from finite data may lie in the unstable (divergent) region of the parameter space. In this way, the simulated model would show qualitatively different dynamics than the underlying data set from which it was derived (i.e., it would diverge).

Another potential reason is the extent of model misspecification. Constraining the true data-generating process into the form of the nonlinear Hawkes process, as assumed here, might bias the parameter estimation towards the parameter space for which model dynamics is unstable. An example for such model misspecification are non-stationary firing rates. In the present models, we assumed a constant baseline firing rate. Maximum-likelihood estimates have shown to be systematically biased if the non-stationarity is not accounted for in the model [37]. Detecting and accounting for non-stationarity in neuronal spiking data is, however, a nontrivial task. The stability framework proposed here can be used as a tool for complementary analyses in this regard.

The quasi-renewal approximation can be used alongside other goodness-of-fit tests to assess model adequacy. It would be a reasonable demand for a model to be classified as stable or fragile with high expected divergence times if it is to be used within the context of simulations or closed-loop applications. Even outside the context of simulations, a model classified as divergent is failing a goodness-of-fit test as it is not able to reproduce the statistics of the training data. Conversely, a model classified as stable might produce finite steady-state firing rates but may still fail with regards to other statistics of the training data, such as first- and second-order firing statistics, interval correlations, or burst properties. Our work exemplifies that it is important to check goodness-of-fit using a comprehensive battery of tests to check for different types of model misspecifications.

The derivation of the quasi-renewal approximation involves a series expansion which here we truncated after the first order. As it was previously stated, this approximation becomes less valid with non-vanishing second- and higher-order spike-spike correlations [26]. This is the case, e.g., for strongly bursting neurons. We have observed these limitations in the exploration of the parameter space over which the QR approximation is valid (Figs 5 and 7). In principle, second- and higher-order correlation terms can be included in the expansion. In particular, Naud et al. [26] truncate the series after the second order. In this case, a self-consistent solution has to be found in terms of the steady-state firing rate and the steady-state spike train auto-correlation.

An additional limitation is inherent to the reduction of the spiking dynamics to a one-dimensional description of a transfer or gain function. For high firing rates, spike trains are constrained by the absolute refractory period and exhibit strong regularity. The transfer function can be qualitatively different when point process histories are assumed to be periodic spike trains of a particular rate rather than homogeneous Poisson-like firing with vanishing higher-order correlations. Hence, the description of the dynamics using a single transfer functions becomes less valid for high firing rates. In practice, this happens when activity diverges beyond which the usefulness of the QR approximation becomes limited.

### Relaxing assumptions and extensions

We now discuss possible relaxing assumptions and extensions to the quasi-renewal framework to assess stability and dynamics of neural point process models.

First, the ability to separate the effect of the most recent spike from all previous spikes and to use the moment-generating functional are unique to the exponential nonlinearity. Possibly, for other specific choices of the nonlinearity a similar manipulation or approximation of Eq (11) may be conceivable, or the firing rate transfer function may be estimated by numerical simulation of the model. However, we consider such an extension of the theory beyond the scope of the present work. Using a different nonlinearity will likely require to invoke some type of Lipschitz condition and alternative ways of studying the stability properties (see, e.g., [9]).

Second, the nonlinear Hawkes process can be formulated as a multivariate process to describe an ensemble of coupled neurons [4, 5, 21]. The corresponding extension of the quasi-renewal framework is possible [27] and may be used to study stability of such networks of heterogeneous neurons. Local linear stability analysis of derived fixed points for the neuronal network dynamics can then be readily implemented based on the spectral radius of a coupling matrix (obtained from coupling coefficients, history filters’ integrals, and the nonlinearity’s first derivative) computed at the fixed point locations [9, 21].

Third, we assumed no (time-varying) exogenous input. The framework can be easily extended to accommodate non-stationary inputs, such as stimulus drive, by allowing the baseline firing rate *c* in Eq (3) to be time-dependent and performing the QR stability analysis for the supremum of *c* as long as such a bound exists. If stability is predicted for this dominating model, the model with time-varying exogeneous input will be stable as long as the exogeneous drive is independent of the firing rate of the neuron itself [21]. This generalization allows the stability analysis to be performed, e.g., for state-space models such as linear dynamic systems with conditionally Poisson observations, where spike-history effects are combined with neural couplings to a low-dimensional latent state whose dynamics is stable itself [2, 38–42].

Fourth, throughout this study, we assumed an absolute refractory period of 2 ms. Because the absolute length of such a refractory period does not impact the theoretical analysis, a refractory period of arbitrary length may be used. Although the existence of refractory periods is pervasive in most physical applications, the assumption of an absolute refractory period might not be justified in some cases. The refractory period leads to a finite support of the transfer function and a bounded firing rate. In the limit of a vanishing refractory period, there might be additional alignments of fixed points to those outlined in Fig 3, such as a single stable and a single unstable fixed point, which can be similarly classified into stable, fragile, and divergent dynamics.

Finally, we provided an approximation of the divergence time for fragile, metastable models. A more direct estimation of the divergence time seems desirable. One alternative to estimate the divergence time would be a fluctuation analysis in analogy to Brownian motion in a potential. In this case, the divergence time would correspond to the escape time of the particle from the potential given around the low-rate stable fixed point. In first order, noise in this process may be described by white noise, scaled with an intensity that should be proportional (if not equal) to the square root of the rate (Poisson statistics). A more insightful or useful estimate of the fluctuations may also be derived from the power spectral density of the activity in the metastable state [27, 43]. Alternatively, a periodic perturbation approach as in [7] may be attempted to calculate the time scales of expected divergences.

### Alternative stabilization methods

We provided a proof-of-principle of how our proposed quasi-renewal framework can be incorporated into a maximum-likelihood parameter estimation procedure in order to guarantee the stability of models estimated from physiological data. We saw that the ordinary MLE provided unphysiological spike trains while the stabilized version matched first- and second-order statistics of the training data with only marginal loss in predictive power and goodness-of-fit scores.

In our current implementation, we maximize the likelihood of the model under the constraint that the model is predicted to be stable by the QR approximation. The determination of stability based on the number and location of fixed points—essentially a bifurcation analysis—consists of multiple steps and is highly nonlinear. We were not able to differentiate the penalty term to exploit more efficient gradient-based optimization schemes. However, empirically, we have observed that the space of admissible parameters seems to form a single connected, possibly even convex, set. From a computational point of view, the determination of stability involves one-dimensional (scalar) arithmetic that allows fast evaluation of many candidate parameter sets during the optimization procedure. If necessary, additional speed-ups could be obtained by parallelizing the computation of the penalty term when evaluating different local search directions.

We restricted the attainable parameter space to all models that are classified as stable, thereby explicitly excluding fragile models with long expected divergence times. In practice, stable models and fragile models are both candidates for physiological dynamics as long as simulation times are shorter than typical divergence times. Therefore, a more refined cost function than the one used here (see Eq (35)) could involve a penalty proportional to the expected divergence rate E[*T*_div_]^−1^, weighted by a corresponding regularization parameter. The calculation of the expected divergence rate is more computationally expensive than the determination of stability itself but would provide a continuous and potentially smooth penalty function that could be superior to the all-or-nothing penalty term of the proposed optimization scheme.

Alternatively, a computationally efficient regularization is the L1-regularized maximum-likelihood estimate. It provides a convex optimization problem that can be efficiently solved [44–46]. For stronger regularization parameters, estimated coefficients tend towards zero. Nonlinear Hawkes processes with vanishing spike-history filter (coefficients tending towards zero) are always stable. This implies the existence of an optimally L1-regularized solution that is predicted to be stable. Therefore, strong L1-regularization might be an alternative approach to model stabilization.

In addition, a more parametric description of the spike-history kernel could facilitate stability. For example, the addition of an adapting (inhibitory) power-law component is likely to prevent any runaway-excitation and has been observed experimentally under certain conditions [17]. A parametric form like this will have to be accompanied by model selection and appropriate goodness-of-fit tests.

A simple way to ensure stability of nonlinear Hawkes processes in simulations is to implement a “reset condition” following each spike when the previous spiking history is forgotten. This leads to a renewal process with a well-defined stationary and unique solutions [47]. However, by definition, second-order statistics such as ISI correlations cannot be reproduced and spike trains generated with this condition are not realizations from the original nonlinear Hawkes model. Nevertheless, it might be an easy fix in certain applications where stable network simulations are desired without explicitly reproducing physiological spike train statistics [34].

Finally, we note that while previous work in the mathematical domains covers uniqueness and existence theorems for stationary point processes [9, 21], it does not provide predictions of dynamics, metastability, or whether steady-state rates are in a physiological regime. In the case of Lipschitz-continuous nonlinearities, existing conditions for stability are overly conservative and of little relevance for neurophysiologically plausible spike-history filters. We are currently working on relaxations of these conditions that would allow easier characterizations of stability of neuron models and neuronal networks, and we hope to report such results in the future.

### Importance of stable point process models for applications

The ability to predict stochastic stability of a given point process model has merit in its own right and is therefore a main contribution of our study. While stability in simulations is neither a necessary nor sufficient condition for the model fitting procedure itself, and a trade-off between stability constraints and other standard regularized MLE approaches should be considered case by case, data-driven models that are guaranteed to be stable are of major importance for many applications. We will conclude by giving a few examples.

First, to understand brain circuits may mean to be able to rebuild them using artificial components. PP-GLMs offer a direct and relatively well-understood method to derive neuron models from data. But their use in rebuilding brain circuits in simulation is limited if stochastic stability is uncertain. For the simple example of a PP-GLM fit of a neuronal network with an embedded “synfire chain”, Zaytsev et al. ensured stability of the network by adding a reset mechanism of the membrane after each spike to prevent run-away excitation [34].

Second, PP-GLMs are used in the context of (closed-loop) brain machine interfaces. Present-day experiments may interface brain tissue to virtual actuators that in turn provide feedback signals to the brain (e.g., [48]). Future applications of such technology may replace damaged neural tissue by simulated neural circuits which are connected bidirectionally to the brain. Such circuits could potentially be made using PP-GLMs or related models, fitted to the system that is being substituted. Stability of the model is essential in this case to exclude major system malfunction.

A final application is to make spike-timing predictions from neuron models: Given parallel recordings of neuronal activity, precise timing of single spikes can already be predicted using PP-GLMs [5]. However, such predictions have been limited to the very near future (on the order of milliseconds), but typically cannot be used for extended time differences into the future because of model instability. Ensuring stability of PP-GLMs potentially extends the time horizon of spike timing prediction. This may enable applications in epilepsy treatment, seismology, finance, and other fields that study self-exciting dynamical processes.

## Materials and methods

### Ethics statement

For the non-human primate data, all procedures were in accordance with Brown University Institutional Animal Care and Use Committee approved protocols and the Guide for the Care and Use of Laboratory Animals. Approval for the human studies was granted by local Institutional Review Boards (Partners Human Research Committee, Massachusetts General Hospital, Harvard Medical School), and the participant was enrolled after obtaining informed consent.

### Experimental details

Full experimental details for the electrophysiological data from the crab can be found in [6], for the non-human primate data in [31], and for the human data in [30, 49].

### The nonlinear Hawkes process

The nonlinear Hawkes process is a flexible class of self-exciting and/or self-inhibiting point process models [9]. For a stochastic point process, the conditional intensity function is given by [10]:

$$\lambda \left(t|H_{t}\right)=\lim_{\Delta \to 0}\frac{P(\text{spike}\;\text{in}\left(t,t+\Delta \right]|H_{t})}{\Delta },$$ (9)

where $H_{t}$ is the history of the process (i.e., all *K* spikes at times *t*_*k*_ up to time *t*).

In the nonlinear Hawkes model, $\lambda (t|H_{t})$ is assumed to take the following form:

$$\lambda \left(t|H_{t}\right)=\phi \left([\eta *S](t)\right)=\phi \;\left(\sum_{k=1}^{K}\eta \left(t-t_{k}\right)\right),$$ (10)

where $\phi \left(x\right)\;:\;R\to R^{+}$ is a nonlinearity that maps the convolution of the spike train *S* with a causal auto-history kernel η(s)∈R onto a non-negative conditional intensity $\lambda (t|H_{t})$.

Here, we consider *ϕ*(*x*) = *c* exp(*x*) = exp(*I*_0_ + *x*) with *c* = exp(*I*_0_) > 0. The exponential nonlinearity implies that modulations from previous spikes interact in a multiplicative way [50]. The choice for the exponential nonlinearity has both theoretical [2, 7] and empirical support, e.g., from electrophysiological experiments [51, 52]. We only consider the single-neuron (univariate) case although nonlinear Hawkes processes can be similarly defined for coupled neuronal ensembles with the corresponding matrix of auto- and cross-history kernels *η*_*ij*_(*s*).

Furthermore, we assume constant exogenous input, i.e., *c* ≡ *const*. For time-varying inputs *c*(*t*) or other (potentially non-stationary) exogenous inputs, a conservative stability analysis can be performed by using *c*_sup_ = sup *c*(*t*) as long as such a bound exists.

Hence, the stochastic process is completely determined by two parameters: *c* (or equivalently *I*_0_) and the causal auto-history kernel *η*(*s*).

To simplify analysis, we make certain assumptions about *η*(*s*). One is the introduction of an absolute refractory period *τ*_ref_ which indicates that the next spike can occur no closer than *τ*_ref_ to the last spike. It can be modeled by setting *η*(*s*) = −∞ for *s* < *τ*_ref_. Given that action potentials, the events that are modeled with the point process model, have an extent of around 1 ms, we assume *τ*_ref_ = 2 ms.

### Quasi-renewal approximation

In the nonlinear Hawkes model, the effects of previous spikes can accumulate. This leads, in general, to a non-renewal process. For this specific class of point process models, there are no closed-form formulas to predict mean intensities, inter-spike interval (ISI) distributions, power spectra, or other properties of the process. We are interested in whether a nonlinear Hawkes process with given parameters permits stable, finite steady-state firing rates.

To make progress, we need to approximate Eq (10). Our approach is based on the recently introduced quasi-renewal approximation [26, 27]. To obtain an estimate of the mean rate of the process *A*_0_, we average $\lambda (t|H_{t})$ over all possible spike trains *S*(*t*) prior to the last spike $\hat{t}$:

$$\begin{array}{ccc} \lambda_{0}\left(t,\hat{t}\right) & = & {\langle \lambda \left(t|H_{t}\right)\rangle }_{S(t<\hat{t})}\\ & = & c\;\exp\left(\eta \left(t-\hat{t}\right)\right)\;{\langle \exp\left(\left[\eta *S\right]\left(t\right)\right)\rangle }_{S(t<\hat{t})}. \end{array}$$

We identify the second term with the moment-generating functional of *S* that can be expanded in a series of moments [26, 53] which we truncate after the first order:

$$\lambda_{0}\left(t,\hat{t}\right)\approx c\;\exp\left(\eta \left(t-\hat{t}\right)\right)\;\exp\left\{\int_{-\infty }^{\hat{t}}\left(e^{\eta (t-t^{\prime })}-1\right)\underset{A_{0}}{\underset{︸}{{\langle S\left(t^{\prime }\right)\rangle }_{S(t<\hat{t})}}}dt^{\prime }\right\},$$ (11)

with $A_{0}={\langle S\left(t^{\prime }\right)\rangle }_{S(t<\hat{t})}$ being the first moment of the averaged spike train, which corresponds to a constant by assuming stationarity. *A*_0_ is the steady-state firing rate which for now remains unknown.

We can rewrite the integration bounds and introduce $\tau =t-\hat{t}$ as the time since the last spike to obtain:

$$\lambda_{0}\left(\tau \right)=c\;\exp\left\{\eta \left(\tau \right)+A_{0}\int_{\tau }^{\infty }\left(\underset{\gamma (u)}{\underset{︸}{e^{\eta (u)}-1}}\right)du\right\},$$ (12)

with *γ*(*u*) = *e*^*η*(*u*)^ − 1 for the exponentiated kernel and $\Gamma =\int_{0}^{\infty }\gamma \left(u\right)du$. Γ is used in the rescaling of the filter for the analysis presented in Fig 9.

Using the quasi-renewal (QR) conditional intensity of Eq (12), we obtain the steady-state survivor function $S_{0}$ and inter-spike interval (ISI) density *P*_0_ as:

$$S_{0}\left(\tau \right)=\exp\left\{-\int_{0}^{\tau }\lambda_{0}\left(u\right)du\right\},$$ (13)

$$P_{0}\left(\tau \right)=S_{0}\left(\tau \right)\lambda_{0}\left(\tau \right),$$ (14)

which, in turn, predict the firing rate:

$$f\left(A_{0}\right)={\left[\int_{0}^{\infty }\tau \;P_{0}\left(\tau \right)d\tau \right]}^{-1}.$$ (15)

Because $\frac{d}{d\tau }S_{0}\left(\tau \right)=-P_{0}\left(\tau \right)$ and integrating by parts:

$$\begin{array}{ccc} f\left(A_{0}\right) & = & {\left[-\int_{0}^{\infty }\tau \frac{d}{d\tau }S_{0}\left(\tau \right)d\tau \right]}^{-1}\\ & = & {\left[-{\left[\tau S_{0}\left(\tau \right)\right]}_{0}^{\infty }+\int_{0}^{\infty }S_{0}\left(\tau \right)d\tau \right]}^{-1}\\ & = & {\left[\int_{0}^{\infty }S_{0}\left(\tau \right)d\tau \;\right]}^{-1}, \end{array}$$ (16)

which is continuous and differentiable in *A*_0_.

### Stability based on the transfer function

Eq (16) defines an average input-output mapping for the single neuron, known as transfer or gain function, which maps an assumed mean input rate *A*_0_ to the mean output rate of the process *f*(*A*_0_). The dynamics of the model can be characterised based on the properties of the transfer function. Fixed points of this map, ${\hat{A}}_{0}=f\left({\hat{A}}_{0}\right)$, can be locally stable or unstable. To detect the fixed points reliably, we search for the zero crossings of the function *g*(*A*_0_) = *f*(*A*_0_) − *A*_0_.

A fixed point ${\hat{A}}_{0}$ is locally stable if $g^{\prime }\left({\hat{A}}_{0}\right)=\frac{d}{dA_{0}}g\left(A_{0}\right){|}_{A_{0}={\hat{A}}_{0}}<0$ and unstable if $g^{\prime }\left({\hat{A}}_{0}\right)\geq 0$. Based on the number and location of fixed points of the mean firing rate map (Eq (16)) we can then classify the model (see Results).

### Prediction of divergence rate for fragile models

For fragile (metastable) models, we may ask whether there is an inter-spike interval *x*, which if several spikes occur repeatedly with this interval, causes a divergence of the firing rate. If such an *x* exists, we can compute the probability of this event. Although other routes to a divergent rate are possible, this one yields an explicit value for its rate of occurrence and can be used as a lower bound for the divergence rate of a metastable model.

Let *t*_1_ be the time of a spike of the process. Then the next spike occurs within the interval *x* with probability $1-S(t_{1}+x,\;t_{1})$, where S is the survivor function. We may now iterate this argument to compute the probability that, following *t*_1_, there is a sequence of *K* spikes with intervals smaller or equal to *x*, as:

$$\begin{array}{ccc} p_{\text{reg}}\left(x\right) & = & \left(1-S\left(t_{1}+x,\;t_{1}\right)\right)\left(1-S\left(t_{1}+2x,\;t_{1}+x\right)\right)\ldots \\ & = & \overset{K}{\underset{k=1}{\prod }}\underset{p\left(x,k\right)}{\underset{︸}{\left[1-S\left(t_{1}+kx,\;t_{1}+\left(k-1\right)x\right)\right]}} \end{array}$$ (17)

To evaluate Eq (17) and compute S, we need to approximate the intensity function $\lambda (t|H_{t})$ for the case that up to *t*_1_ we do not have information about the spike history apart from the rate *A*_0_, but from *t*_1_ on it is defined as the regular firing case with spike times *t*_*k*_ = *t*_1_ + (*k* − 1)*x* for *k* ≥ 1. This gives rise to a similar quasi-renewal approximation as for Eq (12):

$$\begin{array}{ccc} \lambda_{\text{reg}}\left(t,x\right) & = & {\langle \lambda (t|H_{t})\rangle }_{S(t<t_{1})}\\ & = & c\text{ exp}\left\{\sum_{k=1}^{\infty }\;\eta \left(t-t_{k}\right)\right\}\;{\langle \text{exp}\left\{\left[\eta *S\right]\left(t\right)\right\}\rangle }_{S(t<t_{1})}\\ & \approx & c\text{ exp}\left\{\sum_{k=1}^{\infty }\eta \left(t-t_{1}-\left(k-1\right)x\right)\right\}\text{ exp}\left\{\int_{-\infty }^{t_{1}}\left(e^{\eta \left(t-t^{\prime }\right)}-1\right)A_{0}dt^{\prime }\right\}\\ & = & c\text{ exp}\left\{\sum_{k=1}^{\infty }\eta \left(t-t_{1}-\left(k-1\right)x\right)\;\right\}\text{ exp}\left\{A_{0}\int_{t-t_{1}}^{\infty }\gamma \left(s\right)ds\;\right\}. \end{array}$$ (18)

Inserted into Eq (17), we then first check whether the sequence *p*(*x*, *k*) increases monotonically towards 1, setting *t*_1_ = 0. If for a given *k* we have *p*(*x*, *k*) > *p*(*x*, *k* + 1), we terminate the iteration because *x* does not seem to lead to the regular divergence and return *p*_reg_ = 0. If, in contrast, for some value of *k*, *p*(*x*, *k*) is close to 1, we have found a divergent case that occurs with probability *p*_reg_(*x*), as given by Eq (17) with *K* = *k*.

This procedure is performed for all $x\in [\tau_{\text{ref}},A_{0}^{-1}]$, and the maximum max_*x*_ *p*_reg_(*x*) is returned. As *p*_reg_(*x*) is the probability of the regular divergence with intervals *x* or shorter to occur after any spike of the process, the rate of divergence is thus bounded from below by:

$$r_{\text{div}}\geq A_{0}\max_{x}p_{\text{reg}}\left(x\right)\;.$$ (19)

This provides an upper bound for $T_{\text{div}}=r_{\text{div}}^{-1}$.

### Bursting and the regular spiking limit

Apart from the dynamic stability that we have discussed so far, a particular limit of the space of possible spike trains is of special interest. In case of a divergent firing rate, which occurs in unstable or fragile models, the analysis of the gain function predicts that the firing rate saturates at the limit given by the inverse of the refractory period, $A_{0}=\tau_{\text{ref}}^{-1}$. However, there is only one spike train that can realize this firing rate, which is the regular spike train:

$$S_{x}\left(t\right)=\sum_{k=-\infty }^{\infty }\delta \left(t-kx\right),$$ (20)

with inter-spike-interval *x* = *τ*_ref_. Here *θ* denotes the Heaviside function and *δ* denotes the Dirac delta function.

For the regular spike train *S*_*x*_, with *x* > *τ*_ref_ being close to the refractory period, to be a possible mode of firing of the model, it is necessary that the conditional intensity of the neuron (Eq (10)), evaluated at time *x* after the last spike (at *t* − *x*),

$$\lambda_{\text{reg}}^{K}\left(x\right)=c\exp\left[\sum_{k=1}^{K}\eta \left(kx\right)\right],$$ (21)

reaches a sufficiently high value, so that the rate *x*^−1^ can be maintained, when the regular spike train extends into the past forever, $\lambda_{\text{reg}}\left(x\right)=\lim_{K\to \infty }\lambda_{\text{reg}}^{K}\left(x\right)$. But what precisely is that sufficiently high value of *λ*_reg_? Since after *τ*_ref_ the refractory period is over, and because the conditional intensity changes approximately on the time scale of the filter *η* that is much greater than the remaining interval *x* − *τ*_ref_, we may approximate the mean output inter-spike-interval of the process by:

$$\mu_{\text{reg}}\left(x\right)=\tau_{\text{ref}}+\lambda_{\text{reg}}^{-1}\left(x\right)\;.$$ (22)

Now we can formulate a condition on the divergent firing model class: If the expected interval *μ*_reg_ in the regular firing case is smaller or equal to the input interval *x*, the regular firing state can be maintained. From Eqs (22) and (21), we obtain the condition:

$$\sum_{k=1}^{\infty }\eta \left(kx\right)\;\geq -\ln\left[c(x-\tau_{\text{ref}})\right]\;.$$ (23)

This condition is instructive in two ways: First, to maintain the interval *x* = *τ*_ref_, the series on the left hand side (LHS) has to diverge to positive infinity quicker than the logarithm on right hand side (RHS). Second, in case the series on the LHS of Eq (23) converges for all *x* ≥ *τ*_ref_, there is a minimum value of *x* for which Eq (23) is still fulfilled. Then *x*^−1^ is the peak firing rate that this model can maintain close to the regular spiking limit.

Many models, even ones with an upper unstable fixed point, might not fulfill Eq (23). These models are fragile, but also cannot maintain the regular firing mode. Nonetheless we may ask for how many regular spikes they can maintain the tonic activity. This can be addressed by a modified condition like Eq (21) considering *K* < ∞. By analogous reasoning as above, we arrive at the condition:

$$\sum_{k=1}^{K}\eta \left(kx\right)\;\geq -\ln\left[c(x-\tau_{\text{ref}})\right]\;.$$ (24)

For a given tonic firing interval *x* close to *τ*_ref_ (e.g., defined as $x^{-1}=0.9\times \tau_{\text{ref}}^{-1}$), the maximum *K*_max_ for which Eq (24) is fulfilled yields a good approximation of the duration *K*_max_ *x* of the intermittent regular spiking episodes of the model.

If the spike-history filter is a sum of two exponential terms, the condition in Eq (24) takes the specific form of a geometric series:

$$\begin{array}{ccc} -\text{ln}\left[c\left(x-\tau_{\text{ref}}\right)\right] & \leq & \overset{K}{\underset{k=1}{\sum }}\eta \left(kx\right)=\overset{K}{\underset{k=1}{\sum }}\left[J_{\text{r}}e^{-kx/\tau_{\text{r}}}+J_{\text{a}}e^{-kx/\tau_{\text{a}}}\right]\\ & = & J_{\text{r}}e^{-x/\tau_{\text{r}}}\overset{K}{\underset{k=0}{\sum }}e^{-kx/\tau_{\text{r}}}+J_{\text{a}}e^{-x/\tau_{\text{a}}}\overset{K}{\underset{k=0}{\sum }}e^{-kx/\tau_{\text{a}}}\\ & = & J_{\text{r}}e^{-x/\tau_{\text{r}}}\frac{1-e^{-Kx/\tau_{\text{r}}}}{1-e^{-x/\tau_{\text{r}}}}+J_{\text{a}}e^{-x/\tau_{\text{a}}}\frac{1-e^{-Kx/\tau_{\text{a}}}}{1-e^{-x/\tau_{\text{a}}}}\\ & = & J_{\text{r}}\frac{1-e^{-Kx/\tau_{\text{r}}}}{e^{x/\tau_{\text{r}}}-1}+J_{\text{a}}\frac{1-e^{-Kx/\tau_{\text{a}}}}{e^{x/\tau_{\text{a}}}-1}, \end{array}$$ (25)

which for *K* → ∞ becomes condition Eq (23), which here is:

$$-\text{ln}\left[c(x-\tau_{\text{ref}})\right]\leq J_{r}{\left(e^{x/\tau_{r}}-1\right)}^{-1}+J_{a}{\left(e^{x/\tau_{a}}-1\right)}^{-1}.$$ (26)

The boundary defined by Eq (26) in the (*J*_*r*_, *J*_*a*_) space is a line: For every *J*_*r*_ there is a maximum $J_{a}^{\text{max}}$ from which on Eq (26) is true. For $J_{a}<J_{a}^{\text{max}}$, in contrast, we are assured that the regular firing mode with interval *x* is unstable. Models for which Eq (26) is not fulfilled will show intermittent bursting activity (compare with Fig 7).

### Estimation of average divergence time from simulations

We estimate the average divergence time of a given neuron model by simulating *N* = 48 independent neurons for *T* = 1000 s each. A neuron is said to have diverged at time *t* if its average firing rate in the interval [*t* − 1, *t* + 1] seconds exceeds $\lambda_{thr}=0.9\times \tau_{\text{ref}}^{-1}$. Alternatively, the firing rate may stay below *λ*_*thr*_ until the end of the simulation (“censored observation”).

We can now derive the maximum-likelihood estimate of the divergence time. We assume that neurons diverge randomly with rate *r* = 1/*T*_div_. This seems to be justified in practice based on our simulations. Then, the likelihood of observing a divergence time *y* smaller than *T* is $\frac{1}{T_{\text{div}}}e^{-y/T_{\text{div}}}$ and the probability to observe a censored observation of length *T* is given by:

$$\int_{T}^{\infty }\frac{1}{T_{\text{div}}}e^{-y/T_{\text{div}}}dy=e^{-T/T_{\text{div}}}\;.$$ (27)

If we denote the observed divergence times as *y*_1_, *y*_2_, …, *y*_*k*_, and we have *N*_*c*_ = *N* − *k* censored observations, the overall log-likelihood function is given by:

$$\log L=\sum_{i=1}^{k}\log\left(\frac{1}{T_{\text{div}}}e^{-y_{i}/T_{\text{div}}}\right)+N_{c}\log\left(e^{-T/T_{\text{div}}}\right)$$ (28)

$$=-\left(N-N_{c}\right)\log\left(T_{\text{div}}\right)-\frac{N_{c}T}{T_{\text{div}}}-\frac{\sum y_{i}}{T_{\text{div}}}.$$ (29)

At the maximal (log-)likelihood estimate ${\hat{T}}_{\text{div}}$, the gradient with respect to *T*_div_ has to vanish:

$$\frac{\partial \log L}{\partial T_{\text{div}}}{|}_{T_{\text{div}}={\hat{T}}_{\text{div}}}=-\frac{N-N_{c}}{{\hat{T}}_{\text{div}}}+\frac{N_{c}T}{{\hat{T}}_{\text{div}}^{2}}+\frac{\sum y_{i}}{{\hat{T}}_{\text{div}}^{2}}\overset{!}{=}0\;,$$ (30)

$${\hat{T}}_{\text{div}}\left(N-N_{c}\right)=N_{c}T+\sum y_{i}\;,$$ (31)

$${\hat{T}}_{\text{div}}=\frac{N_{c}T+\sum y_{i}}{N-N_{c}}.$$ (32)

This assumes that there was at least one non-censored observation (*N*_*c*_ < *N*). Otherwise, we set ${\hat{T}}_{\text{div}}=\infty$. Note that if *N*_*c*_ > 0, ${\hat{T}}_{\text{div}}$ may be larger than *T*.

### Model estimation via maximum-likelihood optimization

If *η*(*s*) in Eq (10) is parameterized through a set of basis functions {*B*_*i*_(*s*)} with linear coefficients {*β*_*i*_}, then *η*(*s*) = ∑_*i*_ *β*_*i*_ *B*_*i*_(*s*). All model parameters $\left\{I_{0},\vec{\beta }\right\}$ can be estimated via the statistical framework of generalized linear models (GLMs) [2]. We discretize the spike train to obtain a series of spike counts *n*_*i*_ in each time window of length Δ = 1 ms. The expected spike count is given by the discrete-time approximation of Eq (10) as E[*n*_*i*_] = *λ*_*i*_Δ. The log-likelihood is then proportional to:

$$\log L\propto \sum_{i}\left(n_{i}\log\left(\lambda_{i}\Delta \right)-\lambda_{i}\Delta \right).$$ (33)

For the estimation of physiologically plausible model parameters (Fig 9), we used 10 raised cosine functions [4] with logarithmically spaced peaks up to 400 ms as basis functions {*B*_*i*_(*s*)} for the spike-history filter. In addition, an absolute refractory period of *τ*_ref_ = 2 ms was enforced. To improve numerical convergence and to ensure finite parameters for very sparse data sets, we added a small L2-penalty term to the log-likelihood function so that the maximum-likelihood estimate (MLE) corresponds to the minimum of the cost function:

$$C\left(I_{0},\vec{\beta }\right)=-\log L\left(I_{0},\vec{\beta }\right)+\alpha \sum_{i=1}^{10}\beta_{i}^{2},$$ (34)

with regularization parameter *α* = 5 ⋅ 10^−4^.

### Stabilization-constrained maximum-likelihood estimation

For the stabilization procedure (Fig 10), we performed the maximum-likelihood estimation (see previous section) under the additional constraint that the model is predicted to be stable by the QR approximation. That is, we optimized the cost function:

$$C\left(I_{0},\vec{\beta }\right)=\left\{\begin{array}{cc} -\log L\left(I_{0},\vec{\beta }\right)+\alpha \sum_{i=1}^{10}\beta_{i}^{2} & \text{if}\;\text{the}\;\text{model}\;\left\{I_{0},\vec{\beta }\right\}\;\text{is}\;\text{predicted}\;\text{to}\;\text{be}\;\text{stable,}\\ \infty & \text{otherwise.} \end{array}\right.$$ (35)

We used a gradient-free optimization algorithm (Nelder-Mead) with a convergence criterion on the change in parameter values ($\frac{∥\Delta \vec{\beta }∥}{∥\vec{\beta }∥}<10^{-4}$).

The initial condition was chosen as the MLE solution for which positive parameter coefficients were set to zero. Because basis functions *B*_*i*_(*s*) are non-negative, this corresponds to a non-positive spike-history filter and ensures that the initial evaluation of the cost function is finite. Then, the optimization starts from a region with finite cost and allows the algorithm to descend to a (local) minimum.

### Simulation

All simulations with the spike-history filter consisting of one or two exponentials (Figs 5 to 8) were performed using NEST [54], with neuron model “pp_psc_delta” in time steps of 0.5 ms. All other spike train simulations were performed with custom-written MATLAB software with a time discretization of 0.2 ms. Analysis and optimization were performed in MATLAB and Python.

## Supporting information

S1 Fig Less-rapidly growing nonlinearities do not prevent instability. (A) In addition to the exponential nonlinearity used in Fig 2 (blue), we also simulated spike trains using two less rapidly growing nonlinearities: First, a linear-rectifier function, i.e., *f*(*x*) = [*x* + 1]_+_ which is *x* + 1 for *x* > −1 and 0 otherwise. The offset is chosen so that the function matches the exponential nonlinearity at *x* = 0 (green). In addition, we used *f*(*x*) = log(1 + *e*^*x*^) (red), i.e., a smooth interpolation between the exponential for small x with linear asymptotic behavior for large *x*. (B) Simulated spike trains for two additional nonlinearities for the two data sets that were shown to diverge in simulations (Fig 2B and 2C).
(TIF)

S2 Fig Same as Fig 7A with *c* = 2 s^−1^. Spike trains are simulated from a nonlinear Hawkes model with fixed baseline *c* = 2 s^−1^ and an auto-history kernel consisting of two exponentials with amplitudes *J*_*r*_, *J*_*a*_, and corresponding time constants *τ*_*r*_ = 0.02 s and *τ*_*a*_ = 0.1 s. Observed divergence times for simulated spike trains are color-coded (same scale as in Fig 5). In the dashed region, no finite divergence times were observed.
(TIF)

S3 Fig Same as Fig 7A with *c* = 10 s^−1^. Spike trains are simulated from a nonlinear Hawkes model with fixed baseline *c* = 10 s^−1^ and an auto-history kernel consisting of two exponentials with amplitudes *J*_*r*_, *J*_*a*_, and corresponding time constants *τ*_*r*_ = 0.02 s and *τ*_*a*_ = 0.1 s. Observed divergence times for simulated spike trains are color-coded (same scale as in Fig 5). In the dashed region, no finite divergence times were observed.
(TIF)
