# Supplementary figures and images for: On the stability and dynamics of stochastic spiking neuron models: Nonlinear Hawkes process and point process GLMs
Source: PLoS Comput Biol. 2017 Feb 24;13(2):e1005390. doi: 10.1371/journal.pcbi.1005390 (PMC5325182; doi:10.1371/journal.pcbi.1005390)

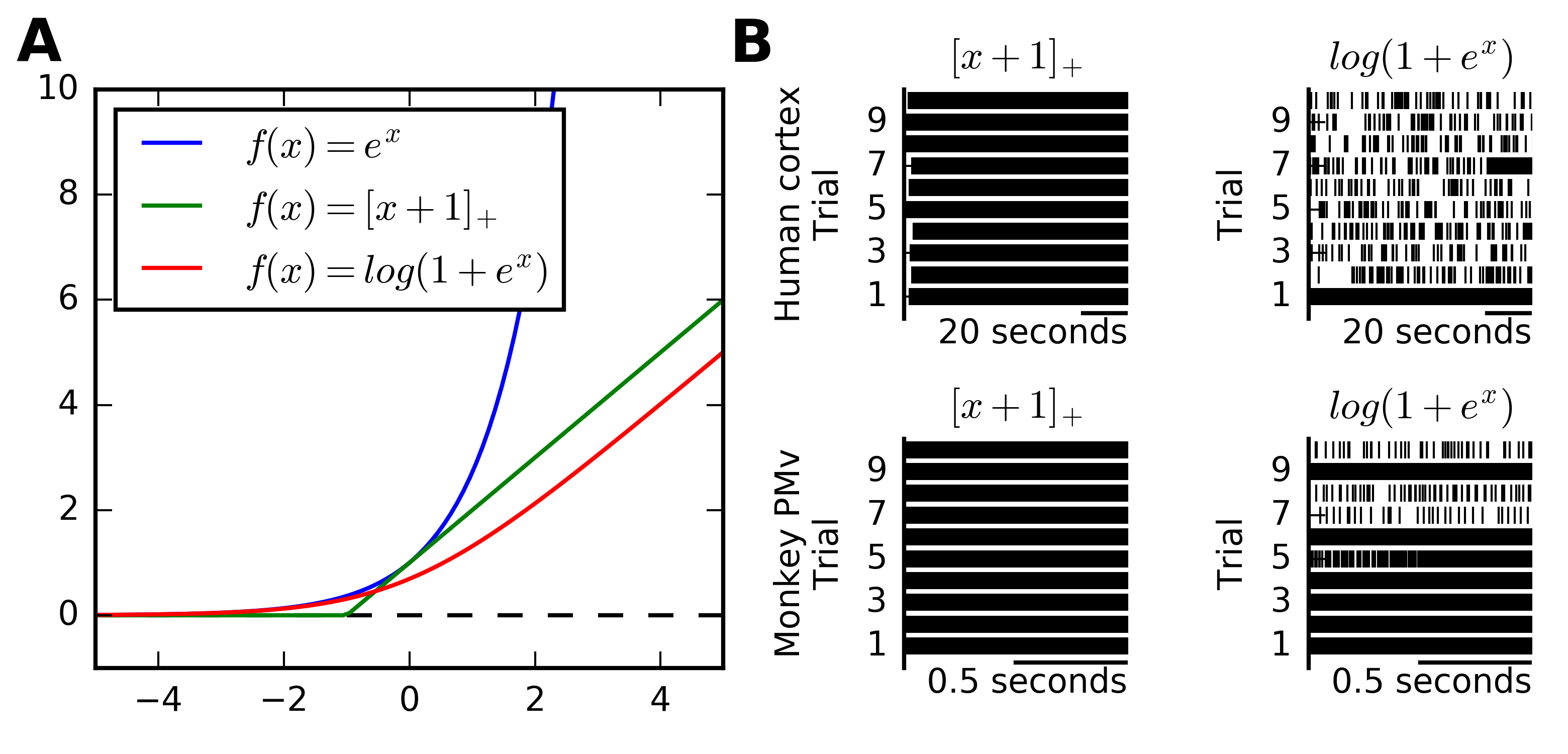

Supplement: S1 Fig — (A) In addition to the exponential nonlinearity used in Fig 2 (blue), we also simulated spike trains using two less rapidly growing nonlinearities: First, a linear-rectifier function, i.e., f(x) = [x + 1]+ which is x + 1 for x > −1 and 0 otherwise. The offset is chosen so that the function matches the exponential nonlinearity at x = 0 (green). In addition, we used f(x) = log(1 + ex) (red), i.e., a smooth interpolation between the exponential for small x with linear asymptotic behavior for large x. (B) Simulated spike trains for two additional nonlinearities for the two data sets that were shown to diverge in simulations (Fig 2B and 2C). (TIF) [file pcbi.1005390.s001.tif]

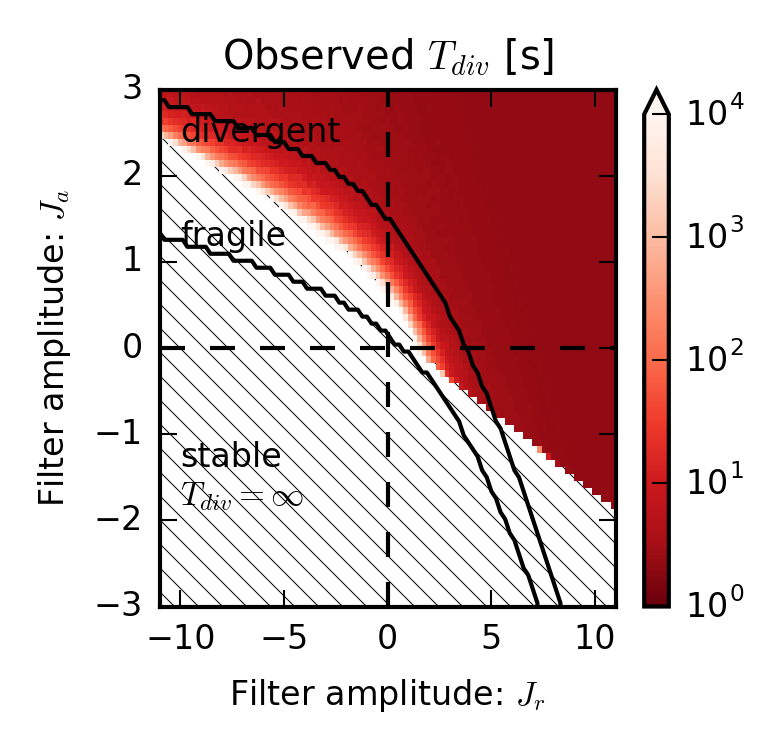

Supplement: S2 Fig — Spike trains are simulated from a nonlinear Hawkes model with fixed baseline c = 2 s−1 and an auto-history kernel consisting of two exponentials with amplitudes Jr, Ja, and corresponding time constants τr = 0.02 s and τa = 0.1 s. Observed divergence times for simulated spike trains are color-coded (same scale as in Fig 5). In the dashed region, no finite divergence times were observed. (TIF) [file pcbi.1005390.s002.tif]

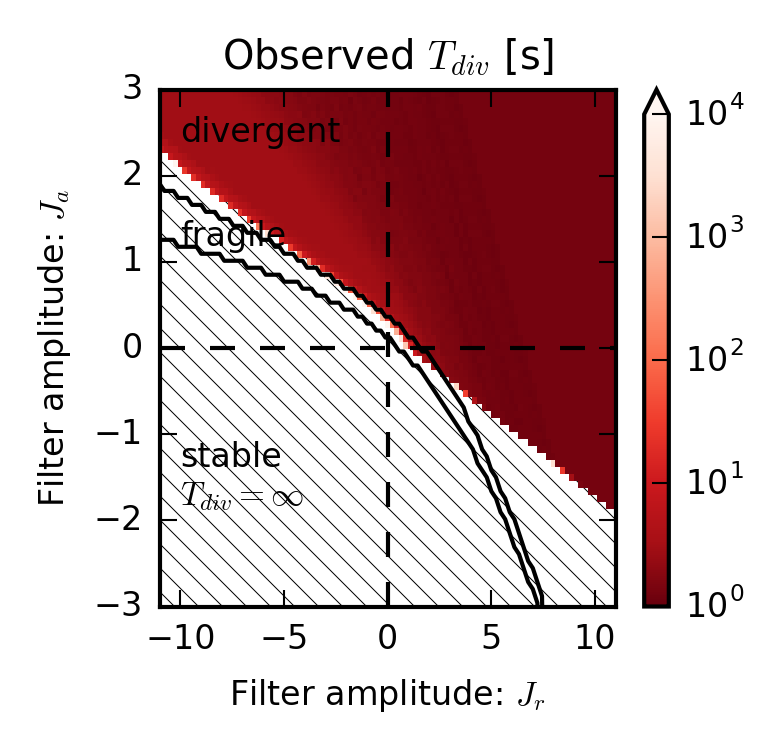

Supplement: S3 Fig — Spike trains are simulated from a nonlinear Hawkes model with fixed baseline c = 10 s−1 and an auto-history kernel consisting of two exponentials with amplitudes Jr, Ja, and corresponding time constants τr = 0.02 s and τa = 0.1 s. Observed divergence times for simulated spike trains are color-coded (same scale as in Fig 5). In the dashed region, no finite divergence times were observed. (TIF) [file pcbi.1005390.s003.tif]
